# In-hospital testing of *NIVPredict* - an AI tool for early prediction of non-invasive ventilation outcome in acute respiratory failure

**DOI:** 10.1186/s13054-026-05894-1

**Published:** 2026-02-15

**Authors:** Hang Yu, Sina Saffaran, Abdisamad Ali, Catherine Henry, Naveed Mustfa, Ajit Thomas, Ashwin Rajhan, Sannaan Isrhad, Liam Weaver, Roberto Tonelli, Luca S. Menga, Qingchen Zhang, Moein Einollahzadeh Samadi, Andreas Schuppert, John G. Laffey, Luigi Camporota, Antonio M. Esquinas, Domenico L. Grieco, Massimo Antonelli, Lucas Martins de Lima, Letícia Kawano-Dourado, Israel S. Maia, Alexandre Biasi Cavalcanti, Enrico Clini, Timothy E. Scott, Declan G. Bates

**Affiliations:** 1 School of Engineering, University of Warwick, Coventry, CV4 7AL UK; 2 NIV Critical Care & Regional Weaning Centre, University Hospital North Midlands NHS Trust, Stoke-on-Trent, UK; 3 Department of Medical and Surgical Sciences of Adult and Mother-Child SMECHIMAI, University of Modena Reggio-Emilia, Modena, Italy; 4 Respiratory Diseases Unit, University Hospital of Modena Policlinico, Modena, Italy; 5 Department of Emergency, Intensive Care Medicine and Anesthesia, Fondazione Policlinico Universitario A. Gemelli IRCCS, Rome, Italy; 6 Istituto di Anestesiologia e Rianimazione, Universita Cattolica del Sacro Cuore, Rome, Italy; 7 Keenan Research Centre, Li Ka Shing Knowledge Institute, St Michael’s Hospital, Unity Health Toronto, Toronto, Canada; 8 Division of Critical Care Medicine, University of Toronto, Toronto, Canada; 9 School of Computer Science and Technology, Hainan University, Haikou, 570228 China; 10 Institute for Computational Biomedicine, University Hospital RWTH, Aachen, Germany; 11 Anaesthesia and Intensive Care Medicine, Galway University Hospitals, Galway, Ireland; 12 Anaesthesia and Intensive Care Medicine, School of Medicine, University of Galway, Galway, Ireland; 13 Intensive Care Medicine, Guy’s and St Thomas’ NHS Foundation Trust, London, UK; 14 Division of Asthma Allergy and Lung Biology, King’s College London, London, UK; 15 Intensive Care Unit, Hospital Morales Meseguer, Murcia, Spain; 16 Hcor Research Institute, Hcor Hospital, Rua Desembargador Eliseu Guilherme, 200 Paraíso, São Paulo, 04004-030 Brazil

**Keywords:** Non-invasive ventilation, Respiratory failure prediction, Machine learning, Decision-support tool, In-hospital testing

## Abstract

**Background:**

Successful non-invasive ventilation (NIV) reduces ICU length of stay, the need for intubation and the risk of death. However, patients who fail NIV and require intubation have a higher risk of death. We developed *NIVPredict*, an easy-to-use web-based AI tool to predict NIV outcome within two hours of initiation in patients with acute respiratory failure (ARF) from diverse aetiologies and tested its useability in a hospital setting.

**Methods:**

This study included data from immunocompromised and immunocompetent patients with hypoxemic ARF due to pneumonia, sepsis or COVID-19, and hypercapnic ARF due to acute exacerbation of chronic obstructive pulmonary disease or obesity hypoventilation syndrome. The tool uses the recently proposed Tabular Prior-Data Fitted Network (TabPFN) machine learning model and was trained using a dataset of routinely collected measurements taken within one hour after NIV initiation in 665 ARF patients from the recent RENOVATE trial in Brazil. Initial external validation of the model was conducted on a dataset of 422 ARF patients from Italy, Spain, and the USA. Subsequently, the useability of a web-based tool based on the model was tested by clinicians at the University Hospitals of North Midlands NHS Trust in the UK between December 2024 and November 2025, who applied it to data collected from 57 eligible ARF patients.

**Results:**

The AI tool provided accurate and robust prediction of NIV outcomes and consistently outperformed conventional clinical indices across all validation settings. In internal repeated cross-validation, external validation, and in-hospital testing, the tool achieved AUCs of 0.793, 0.772, and 0.858, vs. 0.717, 0.709, and 0.693 for the best clinical index (Updated HACOR score), and balanced accuracies of 78.9%, 74.5%, and 85.0%, vs. 68.7%, 63.7%, and 67.6% for the best clinical index (HACOR or Updated HACOR score), respectively.

**Conclusions:**

This study demonstrates superior predictive performance, compared to current clinical indices, of an AI-based tool for NIV outcome prediction on a cohort of patients with overt-acute and acute-on-chronic respiratory failure. Clinical useability of the tool was confirmed via testing by clinicians in a hospital setting, motivating its future evaluation in prospective multi-centre studies.

**Supplementary Information:**

The online version contains supplementary material available at 10.1186/s13054-026-05894-1.

## Introduction

Patients with acute respiratory failure (ARF) who fail non-invasive ventilation (NIV) and subsequently require treatment escalation have a higher risk of death [1–4]. No formal guidelines are currently available to assist clinicians in the early identification of patients at higher risk of NIV failure [4]. Once NIV is initiated, several clinical scores and physiological indices have been proposed to help clinicians predict NIV outcome [5], but significant uncertainty exists regarding their optimal cut-off values and their discriminative power across different datasets or disease aetiologies [6]. In both the widely cited HACOR and Updated HACOR score validation studies [7, 8], and in a recent study using the ROX index [9], important patient subgroups were excluded, i.e. patients with hypercapnic respiratory failure due to chronic obstructive pulmonary disease (COPD) exacerbation or obesity hypoventilation syndrome (OHS), or those who received NIV after failure of high-flow oxygen therapy. These exclusions reduce the applicability of these indices to routine clinical practice in both ward and ICU settings where such conditions are common. Hypoxemic and hypercapnic respiratory failure are distinct entities with different pathophysiology and timing of treatment, and thus it is challenging to develop accurate predictive models that can be applied in both scenarios – a recent study applying the ROX index to data from ARF patients of mixed aetiology produced disappointing results, with the authors concluding that it “cannot currently be recommended for clinical decision support” [10]. Recently, machine learning (ML) models have shown promise to provide more accurate and generalizable predictions of NIV outcome [11], but these models have also only included patients with *de novo* acute hypoxemic respiratory failure, and their clinical useability in a hospital environment has not yet been established.

**Fig. 1:**
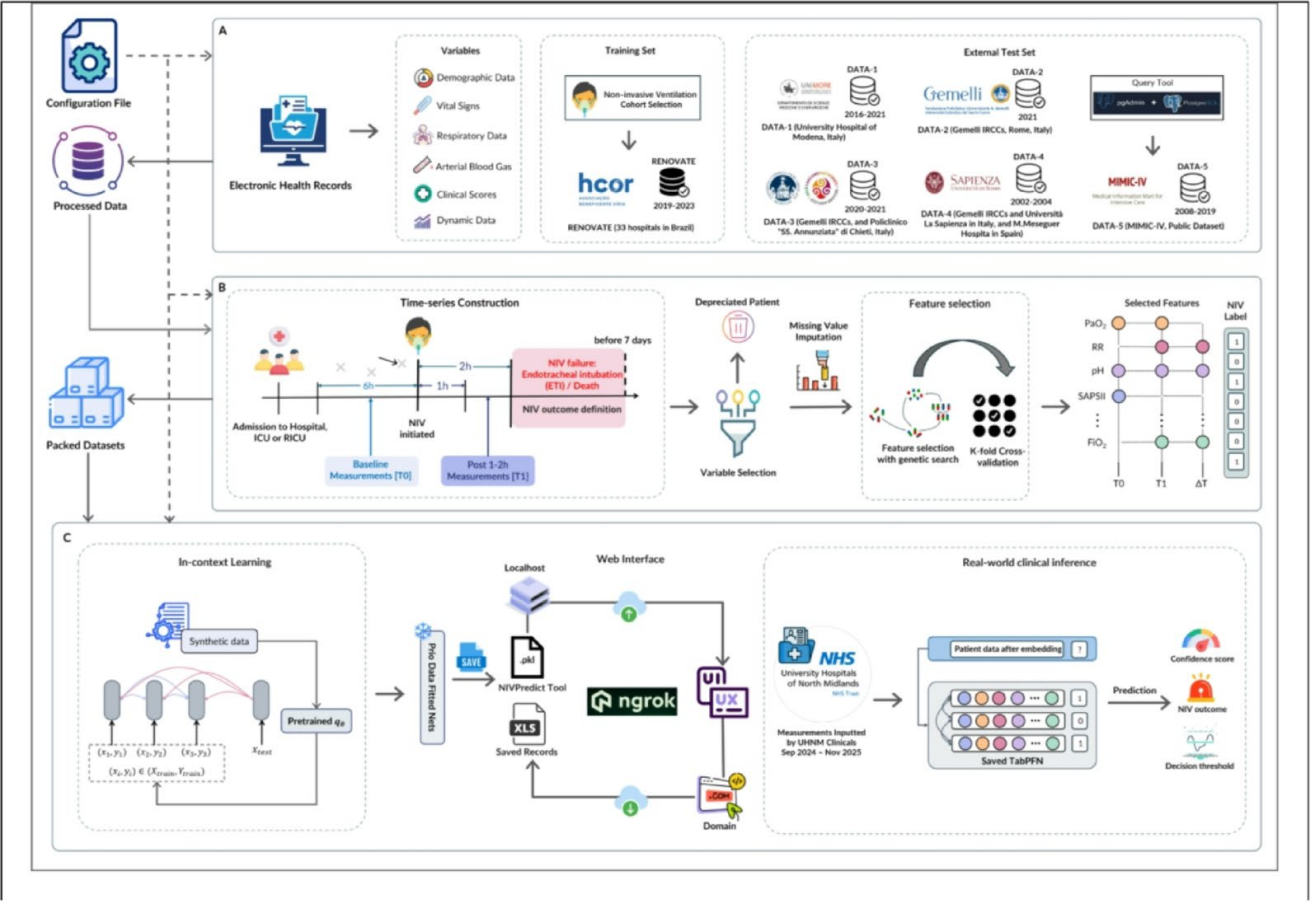
Overview of the AI-driven workflow for NIV outcome prediction and clinical deployment. Panel **A** illustrates the multicentre, retrospective data collection and harmonization across multiple international sources, followed by inclusion/exclusion screening. Panel **B** defines the clinical outcome: NIV failure was defined as endotracheal intubation or death within 7 days after NIV initiation. Input features included baseline measurements within 6 h prior to NIV initiation (T0) and measurements at 1–2 h after NIV initiation (T1). For baseline measurements, when multiple values were available, the one recorded closest to the time of NIV initiation was selected. Missing data were handled through feed-forward processing, and k-nearest neighbour (KNN) imputation strategies. Panel **C** presents the model development phase. Feature selection was conducted on the internal training cohort, and a pretrained TabPFN foundation model was applied using an in-context learning approach. Instead of model retraining, the model leverages synthetic prior knowledge to directly generate predictions based on the encoded inputs. The model performance is evaluated across internal cross-validation and external validation cohorts, using metrics including ROC AUC, net benefit analysis, and calibration curves. When deployed to real-world clinical environment, the *NIVPredict* tool was integrated into a local application using a secure ngrok API and deployed in a hospital setting for testing

In this study, we developed *NIVPredict*, an easy-to-use web-based AI tool, to support clinicians in predicting NIV outcomes across a broad and diverse patient cohort. Model development and reporting followed TRIPOD-AI standards to ensure transparency and reproducibility. We assessed the tool’s accuracy using multiple datasets from different centres and through direct testing by clinicians in a hospital setting (Fig. 1).

## Methods

### Patient data

This was a multicentre, retrospective analysis of prospectively collected data including an in-hospital testing component, conducted across 38 hospitals in four countries (United Kingdom, Italy, Spain, and Brazil), supplemented by data in the publicly available MIMIC-IV dataset from the United States. The study protocol was reviewed and approved by the relevant institutional ethics and research committees at all participating sites. The primary data used for model training via in-context learning and internal cross-validation was taken from the RENOVATE trial [12], which comprises 665 patients (411 successes vs. 254 failures) with ARF who received NIV. The ARF aetiologies in this training set included hypoxemia in both non-immunocompromised and immunocompromised patients, hypoxemic COVID-19, and respiratory acidosis due to acute exacerbations of COPD. Patients diagnosed with cardiogenic, neuromuscular, or traumatic ARF, or interstitial lung disease, were excluded.

For the purposes of external validation, we used a dataset comprising 422 patients (247 successes vs. 175 failures) with ARF who received NIV, compiled from data from previously published studies carried out in Italy and Spain [13–17] as well as from the publicly available MIMIC-IV database from the Beth Israel Deaconess Medical Centre in the United States [18]. The European subset (*N* = 283, 161 successes vs. 122 failures) consisted primarily of acute respiratory distress syndrome (ARDS) and hypoxemic failure secondary to pneumonia, sepsis, and COVID-19, while the US subset (*N* = 139, 86 successes vs. 53 failures) comprised a broader diagnostic mix including COPD, sepsis, pneumonia, and OHS (Additional File: Figure S1 and Table S2).

In-hospital testing of the NIVPredict tool was conducted at the University Hospital of North Midlands NHS Trust (UNHM, UK) between December 2024 and November 2025. The evaluation included 57 patients with ARF receiving NIV in both ward and ICU settings (42 NIV successes vs. 15 NIV failures). The aetiological profile of this cohort included COPD, community-acquired pneumonia (CAP), sepsis, and OHS.

Across all cohorts, physiological measurements were collected at two predefined time points: T0 (baseline values obtained within 6 h prior to NIV initiation) and T1 (values recorded 1–2 h following the start of NIV therapy). NIV failure in all studies was defined by the need for endotracheal intubation or death within 7 days of NIV initiation. 

### Machine learning model

NIVPredict uses the recently proposed Tabular Prior-data Fitted Network (TabPFN) ML model [19]. The software implementing this model is open-source and freely available. In contrast to many ML algorithms that require the availability of very large datasets, TabPFN has been specifically developed for the kind of small-to-medium-sized datasets which are commonly generated in studies in critical care. This new tabular learning method uses in-context learning, the mechanism underlying the unprecedented performance of large language models, and has been shown to significantly out-perform state-of-the-art ML models on small datasets. TabPFN can make predictions without retraining or tuning, even on small or unfamiliar datasets by leveraging knowledge it learned from thousands of synthetic tasks during pretraining. This reduces computational burden and thus eases in-hospital implementation. It also helps reduce overfitting and increases generalizability, thus improving performance on external (unseen) datasets, a critical requirement for any clinical decision support tool. See (Additional File: Methods) for full details of the TabPFN model, including how it was applied in this study.

### Statistical analysis and feature selection

A detailed statistical analysis for each cohort is included in Additional File: Table S1. Feature selection was performed using a genetic algorithm combined with 10-fold cross-validation to automatically identify the most informative features for the machine learning model. These selected features included PaO_2_/FiO_2_ (T1), RR (T1), SAPSII (T0), ΔpH, ΔFiO_2_, PaO_2_/FiO_2_ (T0), PEEP, ΔPaO_2_/FiO_2_, ΔPaCO_2_, PEEP + PSV, ΔRR, COPD_diagnosis, and ICU vs. Ward status. Notably, the model emphasized both static measurements and temporal trajectories in patient status, with some of the most predictive variables being PaO₂/FiO₂ (T1), RR (T1), PaO₂/FiO₂ (T0), ΔpH, and ΔFiO₂. The temporal features capture physiological responses to NIV within the first two hours of treatment, enabling a dynamic assessment that static, single time-point clinical indices usually ignore.

### In-hospital web interface

**Fig. 2:**
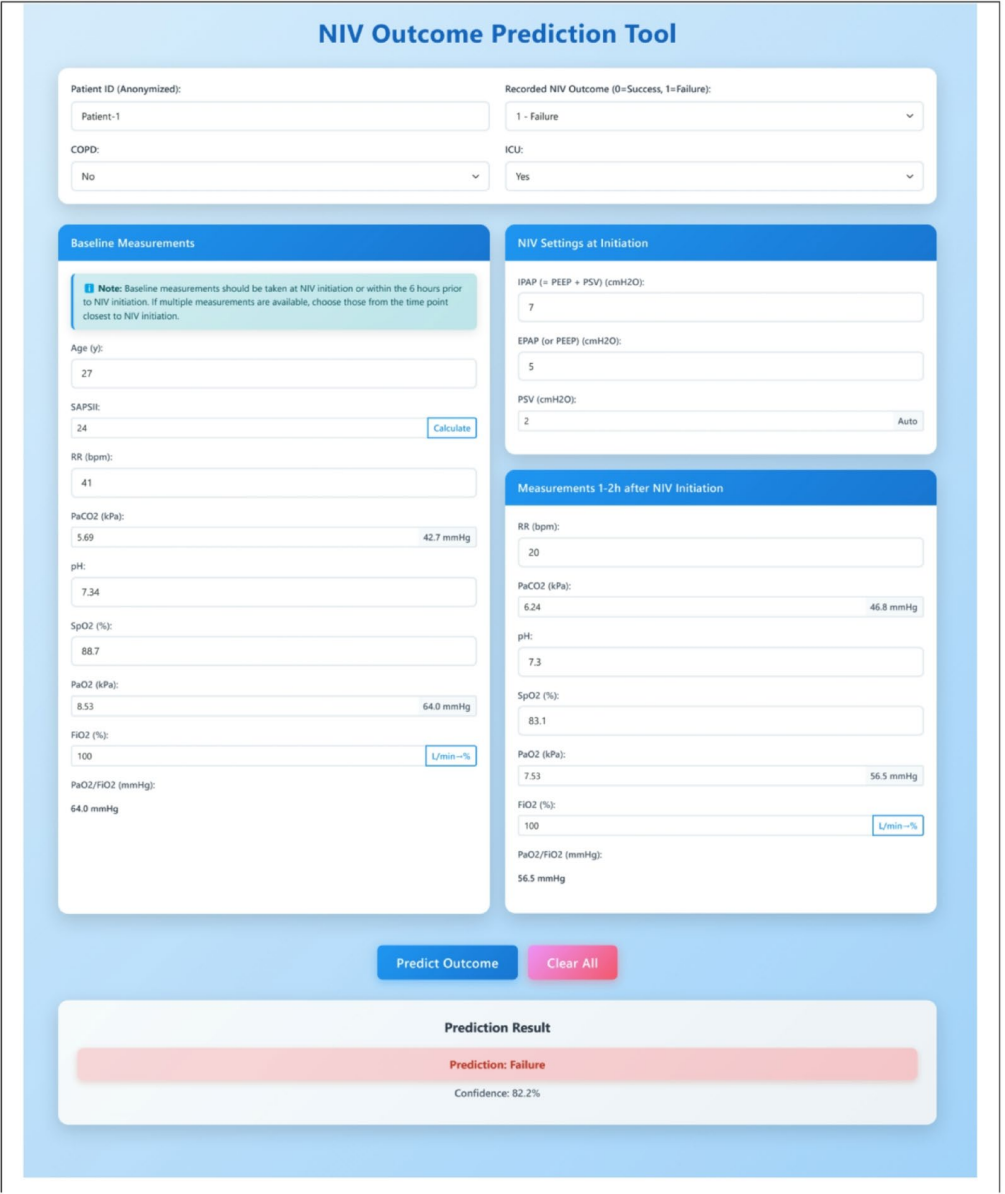
Graphical User Interface of the *NIVPredict* Tool. The interface allows clinicians to enter anonymized patient information, comorbidity status (e.g., COPD), and ICU admission. Baseline measurements within 6 h before NIV initiation and early physiological responses within 1–2 h after initiation are input as model features. NIV settings (IPAP, EPAP, PSV) are also recorded. After data entry, the embedded AI tool generates an immediate prediction of NIV success or failure with a confidence score, enabling rapid clinical decision support at the point of care. No clinical measurements are stored after a prediction is made. Data transmission and storage are handled securely on the local host

A web-based tool, *NIVPredict*, based on the TabPFN model, was developed to enable in-hospital testing by clinicians in a secure data environment. When deployed for in-hospital testing, the *NIVPredict* tool was conditioned only on the internal training dataset. The tool was implemented via Ngrok to allow secure remote access and deployed as a browser-accessible application on local hospital devices. Only the measurements listed on the tool’s graphical user interface, shown in Fig. 2, are required to be entered by the clinician. The confidence score displayed beneath the resulting prediction reflects the probability assigned to the predicted outcome by the TabPFN model. To ensure probabilistic reliability, output probabilities were post-hoc calibrated using Beta calibration [20], based on an independent external validation set.

## Results

### Internal validation

In repeated 5-fold cross-validation on the training dataset, the *NIVPredict* tool achieved a predictive accuracy of 78.2%, sensitivity of 76.8%, specificity of 78.2%, and an AUC of 0.793 (Table 1). The best-performing clinical index in the internal validation was the Updated HACOR score [8] evaluated at timepoint T1, which achieved an accuracy of 68.4%, sensitivity of 69.2%, specificity of 67.4% and an AUC of 0.717.

### External validation

On the multi-centre external dataset, *NIVPredict* attained an accuracy of 74.2%, sensitivity of 76.0%, specificity of 72.9%, and an AUC of 0.772 (Table 1). Decision curve analysis showed that treatment escalation decisions guided by *NIVPredict* provided a greater net benefit than default strategies, such as treating all patients or none, across a wide range of decision thresholds (20% to 70%) (Additional File: Figure S2b). Calibration curves for *NIVPredict*, (Additional File: S2c), closely followed the diagonal reference line, with a Brier score of 0.176 in external validation, indicating strong agreement between prediction confidence and the probability of the prediction being correct. In contrast, clinical indices including HACOR at T1 and SAPS II showed lower predictive performance in external validation, with accuracies of 60.7% and 63.7%, and AUCs of 0.692 and 0.667, respectively. When the HACOR threshold of > 5 originally proposed in [7] was used at T1, performance declined further (Table 1).

**Table 1:** Comparative performance of NIVPredict and conventional clinical indices

**Model**	**Accuracy**	**Balanced accuracy**	**Sensitivity (Recall)**	**Specificity**	**PPV (Precision)**	**NPV**	**AUC**
**Internal validation**							
*NIVPredict*	78.2%	78.9%	76.8%	78.2%	76.5%	79.4%	0.793
HACOR (T0)	59.8%	60.8%	66.2%	55.7%	49.6%	72.3%	0.616
HACOR (T1)	66.5%	67.1%	71.3%	62.5%	55.4%	77.5%	0.697
U-HACOR (T0)	57.7%	60.2%	69.8%	50.3%	47.3%	72.8%	0.634
U-HACOR (T1)	68.4%	68.7%	69.2%	67.4%	57.5%	77.2%	0.717
ROX (T0)	57.2%	59.0%	67.4%	50.7%	46.8%	71.0%	0.612
ROX (T1)	62.7%	64.2%	71.3%	57.1%	52.1%	75.8%	0.691
SOFA	55.2%	54.4%	50.7%	59.1%	44.7%	65.5%	0.591
SAPSII	54.9%	53.2%	45.6%	60.1%	43.7%	63.1%	0.568
**External validation**							
*NIVPredict*	74.2%	74.5%	76.0%	72.9%	70.7%	76.5%	0.772
HACOR (T0) > 4	45.0%	50.6%	83.4%	17.8%	52.0%	31.2%	0.613
HACOR (T1) > 4	60.7%	63.7%	81.7%	45.7%	71.5%	56.5%	0.692
ROX (T0)$\:^\dagger$< 7	50.4%	53.9%	47.2%	60.5%	69.4%	49.1%	0.597
ROX (T1)$\:^\dagger$< 7	59.7%	62.7%	66.0%	59.3%	61.4%	62.2%	0.674
SAPSII > 40	63.7%	61.8%	50.3%	73.3%	60.3%	65.6%	0.667
*NIVPredict* *	76.2%	76.3%	77.3%	75.2%	74.5%	78.0%	0.781
U-HACOR * (T0) > 10	55.8%	56.3%	86.3%	26.3%	66.3%	37.1%	0.640
U-HACOR * (T1) > 10.5	63.2%	63.1%	59.8%	66.4%	77.5%	54.5%	0.709
SOFA * > 4	61.3%	61.1%	49.2%	73.0%	70.7%	56.5%	0.643
**In-hospital testing**							
*NIVPredict*	84.2%	85.0%	86.7%	83.3%	65.0%	94.6%	0.858
HACOR (T0) > 4	42.1%	45.7%	53.3%	38.1%	23.5%	69.6%	0.487
HACOR (T1) > 4	64.9%	67.6%	73.3%	61.9%	40.7%	86.7%	0.685
U-HACOR (T0) > 10	47.4%	60.0%	86.7%	33.3%	31.7%	87.5%	0.518
U-HACOR (T1) > 10.5	57.9%	65.0%	80.0%	50.0%	36.4%	87.5%	0.693
ROX (T0) < 7	54.4%	52.9%	53.3%	52.4%	29.6%	76.6%	0.536
ROX (T1) < 7	63.2%	64.3%	66.7%	61.9%	40.0%	81.3%	0.679
SOFA > 4	75.4%	64.0%	40.0%	88.1%	54.5%	80.4%	0.685
SAPSII > 40	73.7%	58.6%	26.7%	90.5%	50.0%	77.6%	0.717

PPV: Positive Predictive Value, NPV: Negative Predictive Value, AUC: Area Under the Receiver Operating Characteristic Curve. To ensure a fair and comprehensive evaluation, clinical indices were assessed for external and in-hospital validation using thresholds derived from the training dataset using Youden’s J statistic

* Indicates a subset of the original external validation cohort, limited to 269 patients (NIV success: 137; NIV failure: 132) due to missing SOFA score values required for calculating the Updated HACOR score. For details of the optimal thresholds and corresponding performance of each clinical index in each validation cohort, as well as cutoffs reported in the original studies, see Additional File: Table S5, S6

^†^ indicates a subset of the original external dataset, as only MIMIC-IV includes SpO₂ measurements required for calculating the ROX index

In the subset of the external dataset where the Updated HACOR score could be calculated, the *NIVPredict* continued to demonstrate better performance, achieving an accuracy of 76.2% and an AUC of 0.781. While the Updated HACOR score at T1 showed improved performance in this subset, with an accuracy of 63.2% and an AUC of 0.709, its predictive performance remained inferior to *NIVPredict*. Applying the cutoff of > 7 proposed for the Updated HACOR score at timepoint T1 [8] reduced predictive accuracy further (Additional File: Figure S2).

### In-hospital testing

During on-site testing by clinicians at UHNM the *NIVPredict* tool achieved an accuracy of 84.2%, sensitivity of 86.7%, specificity of 83.3%, and an AUC of 0.858. Model calibration was also excellent, with a Brier score of 0.093 (Additional File: Figure S3c). Decision curve analysis showed a greater net benefit across a wider range of decision thresholds (10% to 65%) than for current clinical indices (Additional File: Figure S3b). Restricting predictions to cases where the tool’s confidence score exceeded 60% (*N* = 51/57), increased the tool’s accuracy to 90.2%, sensitivity to 84.6%, specificity to 92.1%, and AUC to 0.859.

In testing at UHNM, where patients were primarily suffering from COPD or OHS, there was a substantial decline in the predictive performance of both the HACOR and Updated HACOR scores (balanced accuracies of 67.6% and 65.0%, and AUCs of 0.685 and 0.693, respectively).

All results given above, broken down according to whether patients had hypoxemic or hypercapnic acute respiratory failure, are included in the Additional File: Tables S7. As shown, superior predictive performance of *NIVPredict* is preserved in both cohorts in all settings. As demonstrated by the separate SHAP analysis for each patient group in the Additional File (Figure S5), the model effectively captures the distinct pathophysiological drivers of NIV failure across different phenotypes. For example, in the hypercapnic cohort, the model assigns significantly greater predictive weight to variables representing ventilatory demand and acid-base status (RR and temporal changes in PaCO₂ and pH), compared to the hypoxemic cohort.

## Discussion

This study presents a novel web-based tool for predicting the outcome of NIV in patients with ARF of diverse aetiologies within the first two hours of treatment. The tool requires only a small number of routinely collected patient measurements to be input via an easy-to-use graphical user interface and can be run as a web application on a smartphone, tablet or laptop. The tool was evaluated using multi-centre retrospective datasets and consistently achieved a level of predictive performance that significantly exceeded that of current clinical scores and indices. In contrast to previous studies that considered only patients with *de novo* acute hypoxemic respiratory failure [11], this new model leverages additional data on 95 patients with hypercapnic respiratory failure due to COPD exacerbation or OHS from the RENOVATE RCT [12] and the MIMIC-IV database [18]. This allowed the development of a more generalizable tool with increased clinical relevance, whose useability by clinicians in a hospital setting could be evaluated for the first time via in-situ testing. Predictions made by the tool can translate into clinical action in two ways. High confidence predictions of NIV success can provide increased confidence that non-invasive support is working and help avoid unnecessary escalation of treatment with attendant risks to the patient and costs to healthcare providers. Conversely, high confidence predictions of NIV failure can prompt clinicians to monitor a patient more closely, reassess current treatment (e.g. adjust pressure settings) or begin planning for treatment escalation. To maximise transparency, no specific risk thresholds are proposed – clinicians should decide for themselves what level of confidence they require from the tool in order to use it to inform their treatment decisions, e.g. to minimize the probability of an incorrect prediction a clinician could decide to disregard any predictions with a confidence level less than 70%.

Our results suggest that the limitations of current clinical scores are not merely due to centre-specific threshold variations, but stem from the inherent lack of discriminative power of static, rule-based indices. Many of these indices are applied at a single time point, ignoring the physiologic trajectory of patient’s responses to NIV that may hold greater prognostic value. Temporal changes such as ΔpH, ΔFiO₂, and ΔPaO₂/FiO₂ which were among the most informative features used by the tool’s TabPFN model (Additional File: Figure S4), reflect the patient’s physiological responses to NIV initiation in a manner not captured by static data-points [3]. This aligns with clinical observations that NIV patients who show improvement in gas exchange and work of breathing tend to have better outcomes [21]. In addition, traditional clinical indices often derive their thresholds retrospectively and usually report only internal validation metrics, limiting their external generalizability and clinical applicability. However, some clinical indices such as HACOR have been developed specifically for patients with *de novo* hypoxemic respiratory failure, and thus their relatively poor performance in this study when applied to datasets from patients with diverse aetiologies (e.g. MIMIC-IV) should be interpreted cautiously as it may be due to population mismatch.

This study has some limitations. Tidal volume, which has been shown to be a predictor of NIV outcome [22, 23], was not included in the measurements input to *NIVPredict* as it was not available in a number of the datasets used for external validation. SAPSII was used as an input to the tool rather than the more easily computed SOFA score for the same reason. A patient’s level of consciousness, degree of cooperation, and fluid balance or volume status are important factors that can impact bedside decision making and success or failure of NIV. In particular, fluid overload may adversely affect gas exchange and respiratory mechanics, especially in patients with cardiac dysfunction or sepsis. Partial information on some of these domains is incorporated into the model through the use of the SAPS II score, which includes variables such as the Glasgow Coma Scale (as a measure of level of consciousness) and urine output. However, while urine output may act as a crude proxy for renal function and volume status, we acknowledge that this does not fully capture fluid balance, nor does it substitute for more granular assessments of volume status (e.g. cumulative fluid balance, echocardiographic parameters, or bioimpedance measures). Unfortunately, more detailed and standardised data on fluid balance or degree of patient cooperation were not available in the datasets used for internal training and external validation of the *NIVPredict* tool, and therefore could not be incorporated into the model. Moreover, several of these variables—particularly cooperation and bedside assessment of volume status—are inherently difficult to quantify in a reproducible manner across different clinicians and healthcare settings. Importantly, *NIVPredict* is intended as a decision-support tool rather than a replacement for clinical judgement. The absence of more granular fluid balance data should not alter the validity of the model or the conclusions drawn, as these factors would be routinely assessed and integrated by clinicians at the bedside alongside the information provided by the tool.

Given that NIV failure trajectories can differ between hypoxemic and hypercapnic patients, the use of phenotype specific assessment intervals beyond the 1–2 h window used here could also be clinically useful and could be incorporated in future versions of the model. Because the hypoxemic group represents a larger proportion of the current training set, the global feature selection process may have been disproportionately influenced by predictors of Type 1 ARF - this cohort imbalance may explain why certain static indicators such as baseline and post 1–2 h PaCO₂ or pH that are likely to be important in hypercapnic patients were not prioritized in the final feature set. Future studies using datasets that incorporate additional factors and allow for more balanced aetiology-specific feature selection processes could allow the model to be re-trained in order to further improve both its predictive accuracy and clinical useability in different populations of ARF patients.

Feasibility/useability assessment was performed via in-hospital testing that took place over one year in a single centre, resulting in a relatively small sample size (*N* = 57). It was observed that the tool achieved higher performance in this test set compared to both internal and external validation cohorts, however this needs to be interpreted with caution as model performance naturally varies with the characteristics of each validation cohort - case mix, disease severity, NIV indications, and local practice (e.g. thresholds for intubation) can all influence model discrimination and calibration. In the external validation cohorts considered here most patients receiving NIV were being managed in the ICU, where NIV is often used in patients at the borderline for intubation. These cases typically involve complex, rapidly evolving conditions, making NIV outcome more difficult to anticipate. In contrast, the in-hospital cohort at UHNM primarily included patients initiated on NIV in the ward setting, where patient selection is more conservative and baseline risk for NIV failure is generally lower. At UHNM, patients with greater clinical uncertainty or who are judged to be at higher risk of deterioration are often intubated directly without a trial of NIV. Hence, there were only a small number of ICU patients in the in-hospital testing cohort (14/57) – these ICU patients generally had a much higher probability of NIV failure and the outcomes in the small cohort are more challenging to predict (Additional File: Table S1). The other main limitation of the study is that it is based on retrospective data. Having established the useability of the tool in a clinical setting, its predictive performance now needs to be fully confirmed in multi-centre prospective studies.

Finally, we emphasise that the proposed tool is designed to be assistive, not prescriptive. Decisions around treatment escalation during NIV are inherently complex and will often be informed by additional considerations that cannot be captured by any set of numbers. In accordance with emerging regulatory frameworks for decision-support systems in healthcare, clinical judgement should always remain the primary arbiter of treatment decisions.

## Conclusions

Using only commonly available measurements taken before initiation and within the first two hours of treatment, the *NIVPredict* tool demonstrated accurate and robust prediction of NIV outcomes in patients with ARF of diverse aetiology, and significantly outperformed the predictive accuracy of currently available threshold-based clinical scores and indices. Practical useability of the tool was confirmed via in-hospital testing by clinicians. These results support the need for future prospective multicentre studies to determine the potential for *NIVPredict* to enhance clinical decision making and improve patient outcomes.

## Supplementary Information

Supplementary Material 1.

## Data Availability

Patient data is available on request from the authors. The *NIVPredict* tool is available for testing as a web-based application - colleagues who would like to evaluate its performance on their own datasets are encouraged to contact Prof. Bates ([d.bates@warwick.ac.uk]).

## Abbreviations

NIV: Non-invasive ventilation
ML: Machine learning
ARF: Acute respiratory failure
ICU: Intensive care unit
COPD: Chronic obstructive pulmonary disease
OHS: Obesity hypoventilation syndrome
CAP: Community-acquired pneumonia
ARDS: Acute respiratory distress syndrome
SHAP: Shapely additive explanation
RR: Respiratory rate
PaO_2_/FiO_2_: The ratio of partial pressure of oxygen in arterial blood to the fraction of inspiratory oxygen concentration
UHNM: University hospital of north midlands NHS Trust
