# Supplementary material for: In-hospital testing of NIVPredict - an AI tool for early prediction of non-invasive ventilation outcome in acute respiratory failure
Source: Crit Care. 2026 Feb 15;30:124. doi: 10.1186/s13054-026-05894-1 (PMC13011697; doi:10.1186/s13054-026-05894-1)
Supplement: Supplementary file 1 — Supplementary Material 1. [file 13054_2026_5894_MOESM1_ESM.docx]

**Additional File**

**In-Hospital Testing of *NIVPredict* - An AI Tool for Early Prediction of Non-Invasive Ventilation Outcome in Acute Respiratory Failure**

Hang Yu^1^, Sina Saffaran^1^, Abdisamad Ali^2^, Henry, Catherine^2^, Naveed Mustfa^2^, Ajit Thomas^2^, Ashwin Rajhan^2^, Sannaan Isrhad^2^, Liam Weaver^1^, Roberto Tonelli^3,4^, Luca S. Menga^5,6,7,8^, Qingchen Zhang^9^, Moein Einollahzadeh Samadi^10^, Andreas Schuppert^10^, John G. Laffey^11,12^, Luigi Camporota^13,14^, Antonio M. Esquinas^15^, Domenico L. Grieco^5,6^, Massimo Antonelli^5,6^, Lucas Martins de Lima^16^, Letícia Kawano-Dourado^16^, Israel S. Maia^16^, Alexandre Biasi Cavalcanti^16^, Enrico Clini^3,4^, Timothy E. Scott^2,*^, and Declan G. Bates^1^

**Affiliations:**

1. School of Engineering, University of Warwick, Coventry CV4 7AL, UK.
2. NIV Critical Care & Regional Weaning Centre, University Hospital North Midlands NHS Trust, Stoke-on-Trent, UK
3. Department of Medical and Surgical Sciences of Adult and Mother-Child SMECHIMAI, University of Modena Reggio-Emilia, Modena, Italy.
4. University Hospital of Modena Policlinico, Respiratory Diseases Unit, Modena, Italy
5. Department of Emergency, Intensive Care Medicine and Anesthesia, Fondazione Policlinico Universitario A. Gemelli IRCCS, Rome, Italy.
6. Istituto di Anestesiologia e Rianimazione, Universita Cattolica del Sacro Cuore, Rome, Italy.
7. Keenan Research Centre, Li Ka Shing Knowledge Institute, St Michael’s Hospital, Unity Health Toronto, Toronto, Canada.
8. Division of Critical Care Medicine, University of Toronto, Toronto, Canada.
9. School of Computer Science and Technology, Hainan University Haikou 570228, China.
10. Institute for Computational Biomedicine, University Hospital RWTH Aachen, Germany
11. Anaesthesia and Intensive Care Medicine, Galway University Hospitals, Galway, Ireland.
12. Anaesthesia and Intensive Care Medicine, School of Medicine, University of Galway, Galway, Ireland.
13. Intensive Care Medicine, Guy’s and St Thomas’ NHS Foundation Trust, London, UK.
14. Division of Asthma Allergy and Lung Biology, King’s College London, London, UK.
15. Intensive Care Unit, Hospital Morales Meseguer, Murcia, Spain.
16. Hcor Research Institute, Hcor Hospital, Rua Desembargador Eliseu Guilherme, 200 Paraíso, São Paulo 04004-030, Brazil

***Corresponding author**: Timothy E. Scott, [Tim.Scott@uhnm.nhs.uk](mailto:Tim.Scott@uhnm.nhs.uk), NIV Critical Care & Regional Weaning Centre, University Hospital North Midlands NHS Trust, Stoke-on-Trent, UK.

Table of Contents

[**Methods** 4](#_Toc219800640)

[**Technical Specifications of the *NIVPredict* Tool** 4](#_Toc219800641)

[**Experimental environments** 6](#_Toc219800642)

[**Tables** 7](#_Toc219800643)

[**Table S1. Characteristics of patients in the different cohorts** 7](#_Toc219800644)

[**Table S2. Overview of multicenter cohorts with aetiological breakdown** 9](#_Toc219800645)

[**Table S3. Cohort definitions and limitations in current available clinical indices** 14](#_Toc219800646)

[**Table S4. Performance comparisons of *NIVPredict*, which uses the TabPFN model, with different standard machine learning models** 15](#_Toc219800647)

[**Table S5. Best cut-off among each cohort for clinical indices excluding hypercapnic respiratory failure and COPD** 16](#_Toc219800648)

[**Table S6. Best cut-off among each cohort for clinical indices on whole cohorts** 17](#_Toc219800649)

[**Table S7. Comparative performance of NIVPredict and clinical indices in hypoxemic and hypercapnic subgroups** 18](#_Toc219800650)

[**Figures** 19](#_Toc219800651)

[**Figure S1. Flow chart of the data extraction process from MIMIC-IV Database.** 19](#_Toc219800652)

[**Fig. S2 ROC-AUC curves, Decision Curve Analysis, and calibration curves for external validation cohort** 20](#_Toc219800653)

[**Fig. S3 ROC-AUC curves, Decision Curve Analysis, and calibration curves for UHNM in-hospital testing cohort** 21](#_Toc219800654)

[**Fig. S4 SHAP summary plot for the *NIVPredict* model** 22](#_Toc219800655)

[**Fig. S5 SHAP feature importance plots for the NIVPredict model applied to hypoxemic and hypercapnic patient cohorts.** 23](#_Toc219800656)

# **Methods**

# **Technical Specifications of the *NIVPredict* Tool**

**The TabPFN Architecture:** TabPFN (Tabular Prior-data Fitted Network) is a transformer-based prior-data fitted network meta-trained on a large distribution of synthetic tabular tasks to approximate Bayesian posterior prediction via amortized inference. At evaluation time, the model receives the training rows (support set) and conditions on them to generate predictions for the test rows; no parameter updates occur during the 'fit' phase while using this model.

The architecture uses self-attention across features, allowing non-linear, conditional interactions (e.g., thresholds and logical gating) while the meta-learned prior provides strong regularization. This enables data-efficient, low-variance predictions even with a small number of inputs. Because the meta-prior encodes common structures observed during meta-training (sparsity, monotonicity, thresholds, interactions), TabPFN can exploit the joint configuration of a few high-signal variables (e.g., P/F ratio, respiratory rate, heart rate) more effectively than additive or shallow models that assume fixed functional forms. Task-specific training or tuning is not performed; performance reflects the coverage of the evaluation task by the meta-prior.

**Feature Selection and Implementation:** The primary design choice optimized for TabPFN in this study was the feature subset. To obtain a compact, high-signal set while avoiding overfitting in a small dataset, a genetic algorithm was utilized within nested cross-validation to search the combinatorial space of subsets. Candidate subsets were scored by inner-fold cross-validation (CV) using the prespecified metric, with a small penalty on subset size to encourage parsimony. The selected subset was locked before outer-fold evaluation and external testing to prevent information leakage. For external validation, the subset was fixed entirely on the development cohort.

the process of using a genetic algorithm (GA) combined with 10-fold cross-validation to perform feature selection for machine learning models. Each chromosome in the population represents a unique subset of features, where each gene (bit) indicates whether a feature is included (1) or excluded (0). The algorithm begins by randomly initializing a population of chromosomes (feature subsets). For each chromosome, a fitness score is calculated by training and evaluating a model using 10-fold cross-validation on the selected features. This ensures that the model’s performance is robust and not dependent on a particular data split. The most promising chromosomes are then selected to form the basis of the next generation. Through crossover (recombination of feature subsets) and mutation (random alterations in selected features), new populations are iteratively generated to explore the feature space efficiently. The process continues until a predefined stopping criterion is met, such as a maximum number of generations or convergence in fitness. This approach is used to automatically identify the optimal combination of features that maximizes model performance, improves generalizability, and reduces overfitting—particularly valuable when dealing with high-dimensional clinical datasets.

**Comparator Models and Metrics:** For comparison machine learning (ML) models requiring hyperparameter tuning, Ray Tune with the Asynchronous Successive Halving Algorithm (ASHA) was employed based on distributed GPUs. The optimization score was set to 'balanced accuracy' (the average of sensitivity and specificity) to mitigate biased predictions due to class imbalance. Operating thresholds were determined on the training set using five-fold CV. Finally, the relative importance of different patient measurements was computed using SHapely Additive exPlanation (SHAP) values, and calibration plots were applied to evaluate the trustworthiness of probabilistic predictions.

**Sample Size Estimation:** For sample size estimation, we used the “pmsampsize” package in R, which yielded a minimum required sample size of 517 patients, including at least 214 events.

# **Experimental environments**

Software and Python Libraries:

All analyses were performed using Python 3.11. The following Python libraries were utilized for data processing, visualization, and machine learning model development:

- Data Processing: pandas, numpy, json, glob, os, itertools, gc, operator, scikit-learn (KNNImputer)
- Statistical Analysis and Visualization: matplotlib, seaborn, scipy, shap, statsmodels, pprint, scipy.stats
- Model Interpretation and Feature Importance: shap, permutation_importance
- Machine Learning Frameworks: scikit-learn, torch, lightgbm, xgboost, joblib, tqdm, PIL, ray
- Utility Libraries: joblib, pickle, operator, itertools, gc, glob, PIL

Computing Resources:

Parallel computing was performed on a system with the following specifications:

- GPU: 1 x NVIDIA Quadro RTX 6000 with 12GB of memory
- CPU: Intel(R) Xeon(R) Gold 5215 CPU @ 2.50 GHz
- CUDA Version: 11.7.0

These resources facilitated efficient computation, particularly in the nested cross-validation of machine learning models.

# **Tables**

## **Table S1. Characteristics of patients in the different cohorts**

| **Variables** | **Training cohort** | |  | **External cohort** | | |  | |  | **UHNM Cohort** | | |  |  |
| --- | --- | --- | --- | --- | --- | --- | --- | --- | --- | --- | --- | --- | --- | --- |
|  | **NIV Failure (N=254)** | **NIV Success (N=411)** | ***P*** | **NIV Failure (N=175)** | **NIV Success (N=247)** | ***P*** | | ***P**** | | **NIV Failure (N=15)** | **NIV Success (N=42)** | ***P*** | | ***P***** |
| **Age, y** | 63 (53, 75) | 64 (53, 76) | 0.754 | 64 (57, 72) | 59 (52,71) | 0.033 | | 0.486 | | 60 (52, 72) | 66 (60, 74) | 0.419 | | 0.362 |
| **COPD, n** | 8 | 34 | 0.008 | 16 | 38 | 0.095 | | 0.399 | | 7 | 25 | 0.545 | | 0.488 |
| **ICU, n** | 218 | 301 | 0.184 | 162 | 204 | 0.003 | | 0.281 | | 9 | 5 | 0.007 | | 0.009 |
| **Baseline measurements (within 6 hours before NIV initiation) (T0 time)** | | | | | | | |  | |  |  |  | |  |
| **RR (bpm)** | 30 (27, 32) | 29 (26, 31) | 0.177 | 31 (26, 35) | 30 (25, 35) | 0.883 | | <0.001 | | 23 (20, 25) | 22 (18, 23) | 0.011 | | <0.001 |
| **pH** | 7.42 (7.40, 7.46) | 7.40 (7.36, 7.45) | 0.022 | 7.39 (7.34, 7.45) | 7.44 (7.40, 7.49) | 0.007 | | <0.001 | | 7.26 (7.23, 7.30) | 7.26 (7.21, 7.33) | 0.771 | | <0.001 |
| **PaO2/FiO2 (mmHg)** | 152 (97, 186) | 173 (108, 192) | <0.001 | 115 (86, 131) | 126 (90, 137) | 0.002 | | <0.001 | | 160 (82, 196) | 184 (150, 223) | 0.072 | | 0.792 |
| **PaO2 (mmHg)** | 79 (60, 87) | 80 (61, 92) | 0.290 | 70 (53, 74) | 72 (59, 77) | 0.042 | | <0.001 | | 82 (60, 80) | 66 (53, 74) | 0.171 | | 0.002 |
| **FiO2 (%)** | 50 (40, 65) | 40 (30, 51) | <0.001 | 65 (55, 70) | 60 (50, 65) | 0.007 | | <0.001 | | 62 (42, 80) | 41 (28, 49) | 0.001 | | 0.003 |
| **PaCO2 (mmHg)** | 38 (31, 41) | 41 (33, 44) | 0.098 | 36 (31, 39) | 37 (31, 40) | 0.657 | | 0.034 | | 68 (59, 71) | 77 (66, 84) | 0.104 | | <0.001 |
| **SAPS II** | 36 (30, 40) | 34 (29, 38) | 0.038 | 37 (31, 46) | 27 (22, 36) | <0.001 | | 0.708 | | 33 (28, 39) | 25 (18, 30) | 0.001 | | <0.001 |
| **HACOR** | 5 (3, 6) | 3 (1, 5) | <0.001 | 9 (7, 9) | 8 (6, 9) | 0.113 | | <0.001 | | 10 (7, 14) | 8 (4, 10) | 0.034 | | <0.001 |
| **1-2 h after NIV initiation (T1 time)** | | | | | | | | | |  |  |  | |  |
| **RR (bpm)** | 28 (23, 30) | 24 (21, 28) | <0.001 | 29 (25, 32) | 27 (24, 30) | 0.015 | | 0.442 | | 23 (20, 25) | 21 (18, 24) | 0.027 | | <0.001 |
| **pH** | 7.41 (7.39, 7.46) | 7.422 (7.40, 7.46) | 0.786 | 7.40 (7.37, 7.43) | 7.41 (7.37, 7.48) | 0.081 | | <0.001 | | 7.30 (7.25, 7.34) | 7.31 (7.27, 7.39) | 0.214 | | <0.001 |
| **PaO2/FiO2 (mmHg)** | 138 (83, 168) | 190 (132, 232) | <0.001 | 146 (101, 168) | 174 (133, 194) | 0.014 | | 0.026 | | 132 (82, 193) | 151 (111, 188) | 0.217 | | 0.001 |
| **PaO2 (mmHg)** | 83 (63, 92) | 88 (68, 103) | 0.009 | 69 (56, 77) | 92 (67, 110) | 0.004 | | 0.837 | | 72 (53, 84) | 58 (54, 63) | 0.049 | | <0.001 |
| **FiO2 (%)** | 60 (40, 70) | 44 (35, 50) | <0.001 | 60 (50, 65) | 50 (45, 60) | 0.007 | | <0.001 | | 62 (50, 80) | 44 (33, 51) | 0.002 | | 0.121 |
| **PaCO2 (mmHg)** | 39 (32, 42) | 39 (33, 43) | 0.725 | 38 (33, 39) | 37 (33, 40) | 0.120 | | 0.749 | | 66 (51, 70) | 91 (55, 74) | 0.318 | | <0.001 |
| **PEEP (cmH2O)** | 8 (7, 9) | 7 (6, 8) | 0.012 | 8 (5, 10) | 8 (5, 10) | 0.546 | | <0.001 | | 5 (5, 6) | 6 (5, 6) | 0.225 | | <0.001 |
| **PSV (cmH2O)** | 10 (8, 12) | 9 (7, 11) | 0.141 | 12 (9, 15) | 13 (10, 15) | 0.007 | | <0.001 | | 11 (10, 12) | 11 (10, 14) | 0.718 | | <0.001 |
| **PEEP+PSV (cmH2O)** | 18 (14, 21) | 17 (12, 20) | 0.031 | 20 (16, 24) | 20 (17, 24) | 0.084 | | <0.001 | | 16 (15, 18) | 17 (14, 20) | 0.431 | | 0.359 |
| **HACOR** | 5 (3, 6) | 3 (0, 5) | <0.001 | 6 (4, 7) | 4 (2, 6) | 0.001 | | <0.001 | | 10 (6, 15) | 9 (1, 12) | 0.891 | | 0.358 |
| **Change in measurements from two time points** | | | | | | | |  | |  |  |  | |  |
| **ΔT0-T1 RR (bpm)** | -2 (-7, 0) | -5 (-8, -1) | 0.007 | -5 (-8, -1) | -6 (-10, -2) | 0.013 | | 0.064 | | -3 (-6, 0) | -1 (-3, 2) | 0.264 | | <0.001 |
| **ΔT0-T1 pH** | -0.01 (-0.03, 0.02) | 0.02 (-0.01, 0.04) | <0.001 | -0.02 (-0.06, 0.01) | -0.01 (-0.04, 0.02) | 0.219 | | 0.166 | | 0.05 (-0.02, 0.06) | 0.05 (0.03, 0.08) | 0.081 | | <0.001 |
| **ΔT0-T1 PaO2/FiO2 (mmHg)** | -4 (-44, 36) | 17 (-42, 75) | 0.037 | 32 (0, 67) | 52 (15, 85) | 0.242 | | <0.001 | | -28 (-49, 28) | -34 (-83, 28) | 0.414 | | 0.002 |
| **ΔT0-T1 PaO2 (mmHg)** | 4 (-10, 21) | 8 (-6, 26) | 0.144 | 13 (-1, 26) | 20 (0, 33) | 0.044 | | <0.001 | | -10 (-10, 11) | -7 (-12, 3) | 0.514 | | 0.002 |
| **ΔT0-T1 FiO2 (%)** | 6 (-2, 14) | 1 (-6, 9) | <0.001 | -5 (-10, 0) | -8 (-16, 0) | 0.092 | | <0.001 | | 0 (-13, 20) | 3 (0, 13) | 0.729 | | 0.107 |
| **ΔT0-T1 PaCO2 (mmHg)** | 1 (-2, 4) | -1 (-4, 3) | 0.020 | 1 (-2, 3) | 0 (-1, 3) | 0.265 | | 0.137 | | -2 (-4, 5) | 13 (-13, -2) | 0.028 | | <0.001 |

Data are presented as median (interquartile range, 25%-75%) for continuous values unless otherwise specified. n for the number of patients. *P* for difference between NIV failure vs. success. *P** for difference between training cohort vs. external cohort. *P*** for difference between training cohort vs. UHNM cohort. Definition of abbreviations: SAPS II = Simplified Acute Physiology Score II. PEEP = Positive end-expiratory pressure. PSV = pressure support value. PEEP + PSV = Inspiratory positive airway pressure (IPAP). HACOR: a clinical index that considers heart rate, acidosis, consciousness, oxygenation, and respiratory rate.

## **Table S2. Overview of multicenter cohorts with aetiological breakdown**

| **Internal: RENOVATE Cohort (33 hospitals in Brazil)** | |
| --- | --- |
| Description | This noninferiority, randomized clinical trial enrolled hospitalized adults (aged ≥18 years; classified as 5 patient groups with ARF: non-immunocompromised with hypoxemia, immunocompromised with hypoxemia, chronic obstructive pulmonary disease [COPD] exacerbation with respiratory acidosis, acute cardiogenic pulmonary edema [ACPE], or hypoxemic COVID-19, which was added as a separate group on June 26, 2023) at 33 hospitals in Brazil between November 2019 and November 2023.  <https://jamanetwork.com/journals/jama/fullarticle/2828065> |
| Inclusion Criteria | Adult patients (aged ≥18 years) were eligible if they were admitted to intensive care units (ICUs), emergency departments, or hospital wards because of acute respiratory failure, which was defined by presence of hypoxemia (oxygen saturation as measured by pulse oximetry [Spo_2_] <90% or Pao_2_ <60 mm Hg at room air) and either respiratory effort (use of accessory musculature, paradoxical breathing, or thoracoabdominal asynchrony) or tachypnea (respiratory rate >25 breaths/min). The extracted cohorts include patients diagnosed with non-immunocompromised hypoxemia, immunocompromised hypoxemia, exacerbation of chronic obstructive pulmonary disease (COPD), or hypoxemic COVID-19, with missing data < 20%. |
| S/F Distribution | 665 patients (NIV success: 411, NIV failure: 254) |
| Aetiology Description | Non-immunocompromised with hypoxemia, immunocompromised with hypoxemia, chronic obstructive pulmonary disease [COPD] exacerbation with respiratory acidosis, or hypoxemic COVID-19 |
| Features Available | ['Age (y)', 'PaO2 (mmHg)', 'PaCO2 (mmHg)', 'pH', 'SpO2 (%)', 'HR (bpm)', 'RR (bpm)', 'PaO2/FiO2 (mmHg)', 'post 1-2h RR (bpm)', 'post 1-2h PaO2 (mmHg)', 'post 1-2h PaO2/FiO2 (mmHg)', 'ROX, 'post 1-2h ROX', 'Bicarbonate', 'SAPSII', 'HACOR', 'post 1-2h HACOR', 'SOFA', 'updated HACOR', 'post 1-2h updated HACOR', 'GCS', 'Systolic blood pressure', 'Temperature', 'Serum sodium', 'Serum urea (BUN)', 'WBC', 'Metastatic cancer', 'Hematologic malignancy', 'FiO2', 'post 1-2h GCS', HR (bpm), post 1-2h HR (bpm), 'PEEP (cmH2O)', 'PSV (cmH2O)', 'post 1-2h SpO2 (%)', 'post 1-2h HR (bpm)', 'post 1-2h FiO2', 'post 1-2h PaCO2 (mmHg)', 'post 1-2h pH'] |
| NIV Treatment Protocol | In the noninvasive ventilation group, the therapy was delivered through a face mask using either a ventilator designed primarily for invasive or noninvasive ventilation. The settings for inspiratory positive airway pressure were between 12 and 14 cm of H_2_O. The expiratory positive airway pressure setting was 8 cm of H_2_O.  Fio_2_ level was titrated to maintain an Spo_2_ within 92% and 98%. |
| Criteria for ETI | (1) respiratory or cardiac arrest; (2) hemodynamic instability with mean arterial pressure lower than 65 mm Hg, systolic arterial pressure lower than 90 mm Hg after proper fluid resuscitation, or need for increasing doses of vasopressors (>0.3 g/kg/min of norepinephrine); (3) cognitive impairment and agitation that prevents medical or nursing care without full sedation; (4) a Glasgow Coma Scale score of less than 11; (5) failure to maintain an Spo_2_ level greater than 92% (or >88% in patients with COPD) despite having an Fio_2_ level of 60%; (6) a progressive increase in Paco_2_ that is greater than 10 mm Hg and a concurrent drop in arterial blood pH level despite attempts of improving ventilation; (7) an intolerability to high-flow nasal oxygen or noninvasive ventilation; (8) development of hypersecretion and inability to eliminate such secretion; (9) require frequent discontinuation of noninvasive ventilation therapy; (10) severe arrhythmia with hemodynamic instability; (11) persistent respiratory acidosis with arterial blood pH level lower than 7.2 after 60 minutes of optimal treatment; or (12) physician discretion. |
| **External: Cohort 1 (single center in Italy)** | |
| Description | This cohort includes data from a prospective observational study conducted in a single eight-bed Respiratory Intensive Care Unit (RICU) at the University Hospital of Modena, Italy, between October 2016 and December 2018, along with data from COVID-19 patients treated in the RICU and ICU of the same hospital between August 1, 2020, and March 15, 2021. The COVID-19 patients were 1:1 propensity score matched with non–COVID-19 patients from the broader dataset spanning 2016 to 2021. All patients were in a comparable phase of acute respiratory failure (ARF), unable to maintain SaO₂ > 92% despite optimized high-flow oxygen therapy, and were therefore candidates for non-invasive ventilation (NIV) according to local clinical protocols. Diagnoses at admission included pneumonia, ARDS, sepsis, or COVID-19.  <https://www.atsjournals.org/doi/10.1164/rccm.201912-2512OC?url_ver=Z39.88-2003&rfr_id=ori:rid:crossref.org&rfr_dat=cr_pub%20%200pubmed> |
| Inclusion Criteria | Patients were age > 18 years; the presence of AHRF with a PaO_2_/FiO_2_ ratio ≤ 220 mmHg, despite high-flow nasal oxygen with the flow set at 60 L/min |
| S/F Distribution | 89 patients (NIV success: 58, NIV failure: 31) |
| Aetiology Description | All patients were in a comparable phase of acute respiratory failure (ARF), unable to maintain SaO₂ > 92% despite optimized high-flow oxygen therapy and were therefore candidates for non-invasive ventilation (NIV) according to local clinical protocols. Diagnoses at admission included pneumonia, sepsis, or COVID-19. |
| Features Available | ['Age (y)', 'BMI (kg/m2)', 'Charlson index', 'Diagnosis 0= pneumonia, 1= ARDS, 2= sepsis/other, 3= COVID-19', 'SAPSII', 'HACOR', 'post 1-2h HACOR', 'SOFA', 'updated HACOR', 'post 1-2h updated HACOR', 'Lactate (mmol/L)', 'Creatinine (mg/dl)', 'RR (bpm)', 'PaO_2_ (mmHg)', 'FiO_2_', 'PaO_2_/FiO_2_ (mmHg)', 'pH', 'PaCO_2_ (mmHg)', 'post 1-2h-RR (bpm)', 'post 1-2h-PaO_2_ (mmHg)', 'post 1-2h-FiO2', 'post 1-2h- PaO_2_/FiO_2_ (mmHg)', 'post 1-2h-PEEP', 'post 1-2h-PSV', 'PEEP+PSV'] |
| NIV Treatment Protocol | Positive end expiratory pressure (PEEP) was initially set at 6 cmH_2_O and subsequently fine-tuned (4–8 cmH_2_O) to target a SaO_2_ > 92% with a delivered FiO_2_ < 70%. Pressure support was set at 10 cmH_2_O and then progressively modified according to VT (VTe/kg of PBW) to target a VTe/kg of PBW, 9.5 ml/kg of PBW and an RR, 30 breaths/min (bpm). |
| Criteria for ETI | 1. PaO_2_/FiO_2_ ratio unchanged or worsened or ,<150 mmHg, 2. the need to protect airways because of neurological deterioration or massive secretions, 3. hemodynamic instability or major electrocardiographic abnormalities, and 4. unchanged or worsened dyspnea and persistence of respiratory distress (RR > 35 bpm, gasping for air, psychomotor agitation 5. requiring sedation, or abdominal paradox). |
| **External: Cohort 2 (single center in Italy)** | |
| Description | The study was conducted at Fondazione Policlinico Universitario A. Gemelli IRCCS in Italy. It included patients admitted to the ICU between March 12 and April 20 with hypoxemic respiratory failure and a confirmed microbiological diagnosis of COVID-19, who were treated with either masked NIV or helmet NIV.  <https://pubmed.ncbi.nlm.nih.gov/33653913/> |
| Inclusion Criteria | Patients were aged >18 years and presented with acute hypoxemic respiratory failure (AHRF), defined by a PaO₂/FiO₂ ratio ≤ 220 mmHg during oxygen therapy administered via an air-entrainment mask at admission, with missing data < 20%. |
| S/F Distribution | 32 patients (NIV success: 12, NIV failure: 20) |
| Aetiology Description | Patients with hypoxemic respiratory failure and a confirmed microbiological diagnosis of COVID-19. |
| Features Available | ['Age (y)', 'BMI (kg/m2)', 'pH', 'PaO_2_ (mmHg)', 'pCO_2_ (mmHg)', 'post 1-2h-PaCO_2_ (mmHg)', 'PaO_2_/FiO_2_ (mmHg)', 'post 1-2h- PaO_2_/FiO_2_ (mmHg)', 'ΔPaO_2_/FiO_2_ (mmHg)', 'PEEP (cmH2O)', 'PSV (cmH2O)', 'post 1/2h-PSV', 'FiO_2_', 'ΔPaCO_2_ (mmHg)', ‘SAPSII’, 'HACOR', 'post 1-2h HACOR'] |
| NIV Treatment Protocol | The specific treatment details are not mentioned. Subjects enrolled in this study were treated with different NIOS methods, but mostly with high PEEP helmet NIV |
| Criteria for ETI | 1. failure to maintain a PaO_2_ 65 mmHg with an FiO_2_$\geq$0.6 with persistent dyspnea, tachypnea, and activation of accessory respiratory muscles; 2. development of conditions necessitating endotracheal intubation to protect the airways (coma or seizure disorders) or to manage copious tracheal secretions; 3. any hemodynamic or electrocardiographic instability (i.e., systemic hypotension lasting >1hr despite fluid resuscitation); 4. inability to correct dyspnea; or inability to tolerate the mask or helmet. |
| **External: Cohort 3 (4 ICUs in Italy)** | |
| Description | Multicenter randomized clinical trial in 4 intensive care units (ICUs) in Italy between October and December 2020, end of follow-up February 11, 2021, including 109 patients with COVID-19 and moderate to severe hypoxemic respiratory failure (ratio of partial pressure of arterial oxygen to fraction of inspired oxygen ≤200).  <https://jamanetwork.com/journals/jama/fullarticle/2778088> |
| Inclusion Criteria | Patients with a PaO₂/FiO₂ ratio ≤ 200 mmHg and PaCO₂ ≤ 45 mmHg, who received ventilation via a helmet interface, were included. Only patients with missing data < 20% were considered for analysis. |
| S/F Distribution | 41 patients (NIV success: 26, NIV failure: 15) |
| Aetiology Description | Patients are primarily diagnosed with COVID-19 |
| Features Available | ['PaO_2_/FiO_2_ (mmHg)', 'PaCO_2_ (mmHg)', 'RR (bpm)', 'FiO_2_', 'post 1-2h-P/F (mmHg)', 'post 1-2h-PaCO_2_ (mmHg)', ‘SAPSII’, 'HACOR', 'post 1-2h HACOR', 'post 1/2h-RR (bpm)', 'post 1/2h-FiO_2_', 'PaO_2_ (mmHg)', 'post 1-2h-PaO_2_ (mmHg)', 'ΔRR (bpm)', 'ΔPaO_2_ (mmHg)', 'ΔFiO_2_', 'ΔPaO_2_/FiO_2_ (mmHg)', 'post 1-2h-PSV', 'post 1-2h-PEEP', 'Age (y)', 'BMI (kg/m2)', 'SOFA', 'PEEP+PSV', 'ΔPaCO_2_ (mmHg), ‘SAPSII’, 'HACOR', 'post 1-2h HACOR'] |
| NIV Treatment Protocol | The ventilator was set in pressure support mode, with the following settings: initial pressure support between 10 and 12 cmH_2_O, eventually increased to ensure a peak inspiratory flow of 100 L/min; positive end expiratory pressure between 10 and 12 cmH_2_O; and FiO_2_ titrated to obtain SpO_2_ between 92% and 98%. Any modification in ventilator settings and interface setup to optimize comfort and patient-ventilator interaction was allowed at the discretion of the attending physicians, but positive end expiratory pressure had to be kept equal to or greater than 10 cmH_2_O. |
| Criteria for ETI | 1. signs of persisting or worsening respiratory failure, defined by at least two of the following criteria: a respiratory rate above 40 cycles/min, lack of improvement of signs of respiratory-muscle fatigue, development of copious tracheal secretions, acidosis with a pH below 7.35, SpO_2_ below 90% for more than 5 min without technical dysfunction, or intolerance to NIV; or one of the following 2. hemodynamic instability defined by a SBP below 90 mmHg, MBP below 65 mmHg or requirement for vasopressor 3. deterioration of neurologic status with a Glasgow coma scale   below 12 points. |
| **External: Cohort 4 (3 ICUs in Italy and Spain)** | |
| Description | All ARDS patients receiving masked NIV and 22 receiving helmet NIV were included. Between March 2002 and April 2004, all consecutive adult patients with early ARDS (defined as occurring within the first 24 hours prior to ICU admission) admitted to three ICUs in Italy (Università Cattolica del Sacro Cuore and Università La Sapienza, Rome) and Spain (M. Meseguer Hospital, Murcia) were considered eligible. All centers applied consistent criteria for selecting candidates for noninvasive positive pressure ventilation (NPPV): a) spontaneous breathing with severe dyspnea at rest; b) respiratory rate ≥ 30 breaths/min; c) diagnosis of ARDS according to the American-European Consensus Conference definition.  <https://pubmed.ncbi.nlm.nih.gov/17133177/> |
| Inclusion Criteria | Patients were age > 18 years; the presence of AHRF with a PaO_2_/FiO_2_ ratio ≤ 220 mmHg, despite high-flow nasal oxygen with the flow set at 60 L/min, with missing data < 20%. |
| S/F Distribution | 121 patients (NIV success: 65, NIV failure: 56) |
| Aetiology Description | Causes of ARDS included sepsis and pneumonia |
| Features Available | ['Age (y)', 'saps II', 'Type of ARDS', 'Causes of ARDS', 'post 1-2h-PEEP', 'post 1-2h-PSV', ' PaO_2_/FiO_2_ (mmHg)', 'post 1-2h-P/F (mmHg)', 'pH', 'post 1-2h-pH', 'PaCO_2_ (mmHg)', 'post 1-2h- PaCO_2_ (mmHg)', 'RR (bpm)', 'post 1-2h-RR (bpm)', 'ΔPaO_2_/FiO_2_ (mmHg)', 'PEEP+PSV', 'ΔRR (bpm)', 'ΔPaCO_2_ (mmHg)'] |
| NIV Treatment Protocol | Pressure support ventilation was increased in increments of 2–3cmH_2_O to obtain an exhaled tidal volume of 6 mL/kg and a respiratory rate of 25 breaths/min. When the helmet was used, part of the volume delivered to the system was spent to distend the helmet and did not reach the patient. PEEP was increased in increments of 2–3 cmH_2_O up to 12 cmH_2_O to ensure a peripheral oxygen saturation of $\geq$ 92% with the lowest FiO_2_ possible. |
| Criteria for ETI | 1. failure to maintain a PaO_2_ 65 mmHg with an FiO_2_$\geq$0.6 with persistent dyspnea, tachypnea, and activation of accessory respiratory muscles; 2. development of conditions necessitating endotracheal intubation to protect the airways (coma or seizure disorders) or to manage copious tracheal secretions; 3. any hemodynamic or electrocardiographic instability (i.e., systemic hypotension lasting >1hr despite fluid resuscitation) 4. inability to correct dyspnea; or inability to tolerate the mask or helmet. |
| **External: Cohort 5 (database, US)** | |
| Description | MIMIC-IV is a publicly available database derived from the electronic health records of the Beth Israel Deaconess Medical Center in the United States. It represents a contemporary dataset, covering a decade of hospital admissions between 2008 and 2019.  <https://www.nature.com/articles/s41597-022-01899-x> |
| Inclusion Criteria | See Fig. S1 for details of data extraction process based on the inclusion criteria. |
| S/F Distribution | 139 patients (NIV success: 53; NIV failure: 86) |
| Aetiology Description | COPD, sepsis, pneumonia, and OHS |
| Features Available | ['PaO_2_ (mmHg)', 'PaCO_2_ (mmHg)', 'FiO_2_', ' PaO_2_/FiO_2_ (mmHg)', 'pH', 'RR (bpm)', 'Age (y)', 'BMI (kg/m2)', 'post 1-2h-PSV', 'post 1-2h-PaO_2_ (mmHg)', 'post 1-2h-PaCO_2_ (mmHg)', 'post 1-2h-FiO_2_', 'post 1-2h-PaO_2_/FiO_2_ (mmHg)', 'post 1-2h-pH', 'post 1-2h-PEEP', 'post 1-2h-RR (bpm)', 'PEEP+PSV', 'ΔRR (bpm)', 'ΔPaO_2_ (mmHg)', 'ΔFiO_2_', 'ΔP/F (mmHg)', 'ΔPaCO_2_ (mmHg)', 'SAPSII', 'HACOR', 'post 1-2h HACOR', 'SOFA', 'updated HACOR', 'post 1-2h updated HACOR'] |
| NIV Treatment Protocol | Unknown |
| Criteria for ETI | Unknown |
| **In-hospital testing:** **UHNM Cohort (UK)** | |
| Description | In-hospital testing of the *NIVPredict* tool was conducted at the University Hospital of North Midlands NHS Trust (UNHM, UK) between December 2024 and November 2025. |
| Inclusion Criteria | Patients were eligible for inclusion if they were admitted to a hospital ward or intensive care unit (ICU) and received NIV as first-line ventilatory support, without prior use of invasive mechanical ventilation (MV). Use of other oxygenation strategies such as supplemental oxygen or high-flow nasal cannula (HFNC) prior to NIV was permitted. Eligible patients were ≥18 years of age and had a diagnosis of acute respiratory failure (ARF), defined as meeting one of the following criteria:  Acute Hypoxemic Respiratory Failure (AHRF): A PaO₂/FiO₂ ratio ≤ 220 mmHg on room air or supplemental oxygen prior to NIV initiation.  Acute-on-Chronic Hypercapnic Respiratory Failure (ACHRF): Defined as either (1) documented chronic respiratory disease (e.g., chronic obstructive pulmonary disease [COPD] or obesity hypoventilation syndrome [OHS]) with evidence of chronic compensation (e.g., elevated bicarbonate), or (2) PaCO₂ > 45 mmHg accompanied by an arterial pH < 7.35, indicating acute decompensation of chronic respiratory failure. |
| S/F Distribution | 57 patients (NIV success: 42; NIV failure: 15) |
| Aetiology Description | COPD, OHS, Community-acquired pneumonia |
| Features Available | ['Age (y)', 'PaO2 (mmHg)', 'PaCO2 (mmHg)', 'pH', 'SpO2 (%)', 'HR (bpm)', 'RR (bpm)', 'PaO2/FiO2 (mmHg)', 'post 1-2h RR (bpm)', 'post 1-2h PaO2 (mmHg)', 'post 1-2h PaO2/FiO2 (mmHg)', 'ROX, 'post 1-2h ROX', 'Bicarbonate', 'SAPSII', 'HACOR', 'post 1-2h HACOR', 'SOFA', 'updated HACOR', 'post 1-2h updated HACOR', 'GCS', 'Systolic blood pressure', 'Temperature', 'Serum sodium', 'Serum urea (BUN)', 'WBC', 'Metastatic cancer', 'Hematologic malignancy', 'FiO2', 'post 1-2h GCS', HR (bpm), post 1-2h HR (bpm), 'PEEP (cmH2O)', 'PSV (cmH2O)', 'post 1-2h SpO2 (%)', 'post 1-2h HR (bpm)', 'post 1-2h FiO2', 'post 1-2h PaCO2 (mmHg)', 'post 1-2h pH'] |
| NIV Treatment Protocol | At UHNM, NIV was delivered according to a standardized, protocol-driven pathway aligned with BTS/ICS guidelines and local adaptations. Treatment was primarily targeted at patients with acute hypoxemic/hypercapnic respiratory failure, particularly those with exacerbations of COPD, OHS, neuromuscular disease (NMD), or chest wall deformity.  NIV was administered using hospital-grade ventilators in S/T (Spontaneous/Timed) mode via a full-face or oronasal mask. Initial settings included:   - IPAP: 10–12 cmH₂O, titrated upward by 2–5 cmH₂O every 10 minutes as tolerated, targeting IPAP 20–30 cmH₂O - EPAP: 4–5 cmH₂O, increased up to 8 cmH₂O as needed - Backup rate: 12–16 breaths/min - FiO₂: Titrated to maintain SpO₂ 88–92% in hypercapnic patients, 92–96% in hypoxemic patients   Therapy was initiated promptly, typically within 60 minutes of confirming persistent acidosis (pH < 7.35, PaCO₂ > 6.5 kPa) following maximal medical therapy. NIV was delivered in designated acute respiratory care areas under continuous monitoring. Repeat arterial blood gases were obtained at 1 hour post-initiation to assess response and guide escalation. |
| Criteria for ETI | Endotracheal intubation (ETI) was considered after 1–4 hours of NIV therapy in patients appropriate for escalation, based on the following clinical criteria:   1. **Worsening respiratory acidosis**: pH < 7.25 or no improvement despite IPAP ≥ 20 cmH₂O 2. **Refractory hypoxemia**: Inability to maintain SpO₂ > 88–92% (or >94% for non-COPD) despite high FiO₂ 3. **Persistent tachypnea**: Respiratory rate > 30–35 breaths/min with ongoing respiratory distress 4. **Neurological deterioration**: GCS < 8 or loss of airway protection (unless reversible CO₂ narcosis) 5. **Hemodynamic instability**: Hypotension requiring vasopressors or presence of severe arrhythmias   A formal review was conducted in cases of clinical deterioration, and the decision to intubate was made based on response to therapy and documented ceiling-of-care plans. |

## **Table S3. Cohort definitions and limitations in current available clinical indices**

| **Indices/Scores** | **Target Population** | **Key Exclusions & Limitations** |
| --- | --- | --- |
| HACOR | Patients with hypoxemic respiratory failure (e.g., pneumonia, ARDS). | - Do-not-intubate (DNI) orders  - Presence of COPD  - Emergency intubation  - NIV intolerance (e.g., refusal due to discomfort) |
| Updated HACOR | Hypoxemic respiratory failure. | - Age < 16 years  - Hypercapnic respiratory failure  - Emergency intubation  - NIV use after planned or accidental extubation  - COPD exacerbation  - NIV following HFNC failure  - NIV started >2 hours before admission to participating center |
| ROX Index | Originally for HFNC failure (pneumonia). | - Not originally validated for NIV pressure support  - Does not account for acidosis (pH). |
| VOX Index | Hypoxemic respiratory failure.  Incorporates Tidal Volume (Vt) as a surrogate for respiratory drive/effort. | - Requires accurate Vt measurement (difficult on some NIV interfaces/HFNC).  - Less validation in hypercapnic cohorts compared to hypoxemic. |
| SOFA | General ICU populations (Sepsis/Multi-organ failure). | - Not NIV specific.  - Slow to change; may not reflect rapid respiratory deterioration or muscle fatigue. |
| SAPS II | General ICU mortality prediction. | - Not NIV specific.  - Static score (calculated at 24h); does not capture the dynamic response to NIV trial (e.g., changes at 1-2 hours). |

## **Table S4. Performance comparisons of *NIVPredict*, which uses the TabPFN model, with different standard machine learning models**

| **ML models** | **AUC** | **Accuracy** | **Sensitivity** | **Specificity** | **PPV** | **NPV** |
| --- | --- | --- | --- | --- | --- | --- |
| *Tabular Learning models* | | | | | | |
| ***NIVPredict* (Training)** | 0.793 | 78.2% | 76.8% | 78.2% | 76.5% | 79.4% |
| ***NIVPredict* (Validation)** | 0.772 | 74.6% | 75.6% | 71.9% | 70.8% | 76.5% |
| *Linear/basic ML models* | | | | | | |
| **SVM (Training)** | 0.731 | 69.4% | 67.5% | 70.2% | 52.3% | 81.6% |
| **SVM (Validation)** | 0.708 | 67.6% | 61.8% | 74.5% | 67.3% | 68.9% |
| **Logistic (Training)** | 0.723 | 70.5% | 68.3% | 71.6% | 53.9% | 81.2% |
| **Logistic (Validation)** | 0.697 | 64.4% | 65.7% | 64.0% | 61.2% | 68.3% |
| **DecisionTree (Training)** | 0.655 | 68.6% | 55.7% | 70.5% | 52.4% | 69.7% |
| **DecisionTree(Validation)** | 0.664 | 60.6% | 56.2% | 64.7% | 58.6% | 63.4% |
| *Ensemble Models* | | | | | | |
| **XGBoost (Training)** | 0.714 | 70.4% | 62.6% | 75.5% | 56.6% | 75.7% |
| **XGBoost (Validation)** | 0.719 | 68.3% | 66.4% | 70.8% | 65.2% | 70.5% |
| **AdaBoost (Training)** | 0.715 | 68.9% | 60.8% | 74.1% | 57.9% | 68.4% |
| **AdaBoost (Validation)** | 0.71 | 67% | 59% | 74% | 66% | 67.0% |
| **GradientBoosting (Training)** | 0.656 | 64.7% | 51.9% | 79.4% | 55.8% | 68.9% |
| **GradientBoosting (Validation)** | 0.708 | 66.6% | 67.5% | 64.8% | 62.3% | 69.4% |
| *Probabilistic Models* |  |  |  |  |  |  |
| **Gaussian Naïve Bayes (Training)** | 0.688 | 66.4% | 50.8% | 76.6% | 58.4% | 70.5% |
| **Gaussian Naïve Bayes (Validation)** | 0.656 | 63.6% | 68.4% | 58.5% | 59.7% | 67.0% |

## **Table S5. Best cut-off among each cohort for clinical indices excluding hypercapnic respiratory failure and COPD**

| **Indices/Score** | **Accuracy** | **Balanced Accuracy** | **Sensitivity (Recall)** | **Specificity** | **PPV (Precision)** | **NPV** | **AUC** |
| --- | --- | --- | --- | --- | --- | --- | --- |
| **Training Cohort** | | | | | | | |
| HACOR (T0) > 4 | 60.6% | 58.2% | 50.6% | 65.8% | 43.9% | 71.6% | 0.610 |
| HACOR (T1) > 4 | 66.5% | 63.3% | 53.0% | 73.6% | 51.6% | 74.8% | 0.679 |
| U-HACOR (T0) > 10 | 58.1% | 60.1% | 66.5% | 53.6% | 43.2% | 75.2% | 0.630 |
| U-HACOR (T1) > 10.5 | 67.3% | 65.2% | 58.2% | 72.2% | 52.5% | 76.5% | 0.695 |
| SAPSII > 40 | 62.3% | 54.6% | 18.6% | 90.6% | 56.1% | 63.3% | 0.595 |
| SOFA > 4 | 55.4% | 54.4% | 49.8% | 59.1% | 44.0% | 64.5% | 0.589 |
| **External Validation Cohort** | | | | | | | |
| HACOR (T0) > 4 | 61.9% | 61.3% | 42.2% | 80.6% | 70.6% | 41.3% | 0.554 |
| HACOR (T1) > 7 | 68.2% | 62.9% | 52.7% | 84.1% | 80.4% | 47.9% | 0.716 |
| U-HACOR (T0) > 13.5 | 54.2% | 59.4% | 36.1% | 82.6% | 76.5% | 45.2% | 0.598 |
| U-HACOR (T1) > 13 | 69.5% | 70.6% | 59.8% | 81.4% | 78.3% | 56.4% | 0.725 |
| SAPSII > 39 | 66.1% | 65.2% | 69.4% | 60.9% | 73.5% | 56.0% | 0.709 |
| SOFA > 3 | 58.2% | 61.0% | 48.2% | 73.9% | 74.3% | 47.7% | 0.684 |
| **In-hospital Testing Cohort** | | | | | | | |
| HACOR (T0) > 10 | 63.2% | 53.6% | 33.3% | 73.8% | 31.3% | 75.6% | 0.487 |
| HACOR (T1) > 9 | 72.0% | 69.5% | 62.5% | 76.5% | 55.6% | 81.3% | 0.772 |
| U-HACOR (T0) > 13.5 | 63.2% | 53.6% | 33.3% | 73.8% | 31.3% | 75.6% | 0.515 |
| U-HACOR (T1) > 10.5 | 72.1% | 75.4% | 85.3% | 65.5% | 63.3% | 586% | 0.785 |
| SAPSII > 24 | 68.0% | 69.8% | 75.0% | 64.7% | 50.0% | 84.6% | 0.783 |
| SOFA > 3 | 68.0% | 63.2% | 50.0% | 76.5% | 50.0% | 76.5% | 0.732 |

The optimal cut-off for each clinical index or score was determined using Youden's J statistic within each cohort. Performance metrics (e.g., sensitivity, specificity, balanced accuracy) were then calculated based on these cohort-specific thresholds. In addition, the area under the ROC curve (AUC) was computed using univariate logistic regression applied to the original dataset. This analysis demonstrates that, even when thresholds are optimized within each cohort, clinical indices alone show limited ability to discriminate NIV outcomes. Moreover, their performance varies considerably across cohorts, and the optimal cut-off values lack consistency, indicating poor generalizability.

## **Table S6. Best cut-off among each cohort for clinical indices on whole cohorts**

| **Indices/Score** | **Accuracy** | | **Balanced Accuracy** | **Sensitivity (Recall)** | **Specificity** | **PPV (Precision)** | **NPV** | **AUC** |
| --- | --- | --- | --- | --- | --- | --- | --- | --- |
| **Training Cohort** | | | | | | | | |
| HACOR (T0) > 4 | | 59.5% | 58.4% | 53.7% | 63.2% | 48.7% | 67.7% | 0.608 |
| HACOR (T1) > 4 | | 61.5% | 61.7% | 62.6% | 60.9% | 51.0% | 71.4% | 0.629 |
| U-HACOR (T0) > 10 | | 57.4% | 59.6% | 70.3% | 48.9% | 47.3% | 71.7% | 0.623 |
| U-HACOR (T1) > 10.5 | | 61.5% | 61.7% | 62.6% | 60.9% | 51.0% | 71.4% | 0.629 |
| SAPSII > 44 | | 61.3% | 53.5% | 16.3% | 90.6% | 53.3% | 62.2% | 0.545 |
| SOFA > 4 | | 54.3% | 53.6% | 50.4% | 56.9% | 43.2% | 63.8% | 0.579 |
| **External Validation Cohort** | | | | | | | | |
| HACOR (T0) > 6 | | 60.3% | 59.1% | 51.4% | 66.8% | 70.3% | 56.1% | 0.613 |
| HACOR (T1) > 7 | | 69.0% | 65.8% | 47.4% | 84.2% | 83.0% | 64.2% | 0.692 |
| U-HACOR (T0) * > 15.5 | | 58.7% | 58.3% | 35.6% | 81.0% | 74.6% | 53.9% | 0.581 |
| U-HACOR (T1) * > 13 | | 65.8% | 65.5% | 47.0% | 83.9% | 81.6% | 59.6% | 0.709 |
| SAPSII > 37 | | 66.4% | 67.0% | 70.3% | 63.6% | 75.5% | 60.6% | 0.721 |
| SOFA * > 2 | | 61.7% | 62.1% | 83.3% | 40.9% | 66.7% | 53.8% | 0.643 |
| **In-hospital Testing Cohort** | | | | | | | | |
| HACOR (T0) > 13 | | 68.0% | 56.6% | 25.0% | 88.2% | 50.0% | 71.4% | 0.526 |
| HACOR (T1) > 12 | | 70.2% | 66.9% | 60.0% | 73.8% | 45.0% | 83.8% | 0.685 |
| U-HACOR (T0) > 16.5 | | 68.0% | 56.6% | 25.0% | 88.2% | 50.0% | 71.4% | 0.585 |
| U-HACOR (T1) > 17.5 | | 68.4% | 67.9% | 66.7% | 69.1% | 43.5% | 85.3% | 0.693 |
| SAPSII > 24 | | 66.7% | 73.1% | 86.7% | 59.5% | 43.3% | 92.6% | 0.765 |
| SOFA > 3 | | 64.9% | 61.2% | 53.3% | 69.1% | 38.1% | 80.6% | 0.694 |

The optimal cut-off for each clinical index or score was determined using Youden's J statistic within each cohort. Performance metrics (e.g., sensitivity, specificity, balanced accuracy) were then calculated based on these cohort-specific thresholds. In addition, the area under the ROC curve (AUC) was computed using univariate logistic regression applied to the original dataset. This analysis demonstrates that, even when thresholds are optimized within each cohort, clinical indices alone show limited ability to discriminate NIV outcomes. Moreover, their performance varies considerably across cohorts, and the optimal cut-off values lack consistency, indicating poor generalizability.

## **Table S7. Comparative performance of NIVPredict and clinical indices in hypoxemic and hypercapnic subgroups**

| **Indices/Score** | **Accuracy** | | **Balanced Accuracy** | **Sensitivity (Recall)** | **Specificity** | **PPV (Precision)** | **NPV** | **AUC** |
| --- | --- | --- | --- | --- | --- | --- | --- | --- |
| ***Hypoxemic ARF Cohort*** | | | | | | | | |
| **External Validation Cohort** | | | | | | | | |
| *NIVPredict* | | 75.3% | 75.2% | 77.4% | 73.0% | 71.2% | 81.1% | 0.781 |
| HACOR (T0) > 4 | | 59.5% | 59.4% | 61.7% | 57.0% | 67.0% | 52.3% | 0.609 |
| HACOR (T1) > 4 | | 66.0% | 65.9% | 68.7% | 63.0% | 73.8% | 58.3% | 0.714 |
| U-HACOR (T0) > 10 | | 56.3% | 53.9% | 88.7% | 19.0% | 67.5% | 29.7% | 0.646 |
| U-HACOR (T1) > 10 | | 65.6% | 65.9% | 61.7% | 70.0% | 74.7% | 83.3% | 0.715 |
| SAPSII > 40 | | 63.7% | 64.8% | 49.6% | 80.0% | 65.5% | 62.5% | 0.658 |
| SOFA > 4 | | 62.8% | 63.5% | 53.0% | 74.0% | 70.1% | 57.8% | 0.647 |
| ROX (T0) $\boldsymbol{\dagger}$ < 7 | | 52.9% | 53.0% | 52.7% | 53.1% | 67.8% | 45.6% | 0.586 |
| ROX (T1) $\boldsymbol{\dagger}$ < 7 | | 63.5% | 64.0% | 66.7% | 61.2% | 64.9% | 62.5% | 0.683 |
| **In-hospital Testing Cohort** | | | | | | | | |
| *NIVPredict* | | 88.0% | 91.2% | 87.5% | 88.2% | 70.0% | 100% | 0.883 |
| HACOR (T0) > 4 | | 40.0% | 55.9% | 100% | 11.8% | 34.8% | 100% | 0.526 |
| HACOR (T1) > 4 | | 56.0% | 64.4% | 87.5% | 41.2% | 41.2% | 87.5% | 0.772 |
| U-HACOR (T0) > 10 | | 56.0% | 61.0% | 75.0% | 47.1% | 40.0% | 80.0% | 0.585 |
| U-HACOR (T1) > 10 | | 76.0% | 76.1% | 87.5% | 70.6% | 53.8% | 91.7% | 0.793 |
| SAPSII > 39 | | 72.0% | 56.3% | 12.5% | 100% | 100% | 70.8% | 0.724 |
| SOFA > 4 | | 68.0% | 60.0% | 37.5% | 82.4% | 42.9% | 77.8% | 0.684 |
| ROX (T0) < 7 | | 60.0% | 60.7% | 62.5% | 58.8% | 38.5% | 83.3% | 0.682 |
| ROX (T1) < 7 | | 68.0% | 63.2% | 50.0% | 76.5% | 50.0% | 76.5% | 0.757 |
| ***Hypercapnic ARF Cohort*** | | | | | | | | |
| **External Validation Cohort** | | | | | | | | |
| *NIVPredict* | | 74.1% | 74.8% | 76.5% | 73.0% | 65.0% | 79.4% | 0.764 |
| HACOR (T0) > 6 | | 64.8% | 53.7% | 23.5% | 83.8% | 100.0% | 62.0% | 0.518 |
| HACOR (T1) > 4 | | 61.1% | 65.3% | 76.5% | 54.1% | 59.1% | 62.5% | 0.662 |
| U-HACOR (T0) > 7 | | 53.7% | 58.3% | 70.6% | 45.9% | 57.1% | 51.5% | 0.627 |
| U-HACOR (T1) > 11 | | 59.3% | 60.8% | 64.7% | 56.8% | 73.3% | 53.8% | 0.655 |
| SAPSII > 34 | | 63.0% | 65.1% | 70.6% | 59.5% | 60.0% | 64.7% | 0.650 |
| SOFA > 3 | | 57.4% | 61.0% | 70.6% | 51.3% | 63.2% | 54.3% | 0.621 |
| ROX (T0) < 6 | | 51.9% | 49.0% | 41.2% | 56.8% | 77.8% | 46.7% | 0.592 |
| ROX (T1) < 9 | | 59.3% | 67.1% | 88.2% | 45.9% | 46.9% | 77.3% | 0.679 |
| **In-hospital Testing Cohort** | | | | | | | | |
| *NIVPredict* | | 81.3% | 82.9% | 85.7% | 80.0% | 66.7% | 90.9% | 0.832 |
| HACOR (T0) > 6 | | 28.1% | 43.7% | 71.4% | 16.0% | 19.2% | 66.7% | 0.434 |
| HACOR (T1) > 4 | | 59.4% | 63.7% | 71.4% | 56.0% | 31.3% | 87.5% | 0.614 |
| U-HACOR (T0) > 7 | | 37.5% | 44.6% | 57.1% | 32.0% | 19.0% | 72.7% | 0.443 |
| U-HACOR (T1) > 11 | | 31.3% | 56.0% | 100% | 12.0% | 24.1% | 100.0% | 0.614 |
| SAPSII > 34 | | 68.8% | 64.6% | 57.1% | 72.0% | 50.0% | 75.0% | 0.717 |
| SOFA > 3 | | 62.5% | 60.6% | 57.1% | 64.0% | 30.8% | 84.2% | 0.606 |
| ROX (T0) < 6 | | 65.6% | 47.1% | 14.2% | 80.0% | 16.7% | 76.9% | 0.694 |
| ROX (T1) < 9 | | 65.6% | 72.9% | 85.7% | 60.0% | 37.5% | 93.8% | 0.714 |

The analysis is stratified into hypoxemic and hypercapnic cohorts to evaluate predictive performance across distinct pathophysiological entities. The hypoxemic cohort primarily consists of patients with pneumonia, sepsis, or COVID-19, while the hypercapnic cohort includes patients with acute exacerbations of COPD or obesity hypoventilation syndrome. Cutoff points for clinical indices were determined using Youden’s J statistic based on the corresponding cohorts in the training set. Data are presented for the External Validation Cohort (Hypoxemic: n=215, failure rate 53.5%; Hypercapnic: n=54, failure rate 31.5%) and the UHNM In-hospital Testing Cohort (Hypoxemic: n=25, failure rate 32.0%; Hypercapnic: n=32, failure rate 21.9%). $\boldsymbol{\dagger}$ denotes a specific subset analysis hypoxemic ARF patients (n=85) which was conducted for variables available only in MIMIC-IV dataset.

# **Figures**

## **Figure S1. Flow chart of the data extraction process from MIMIC-IV Database.**


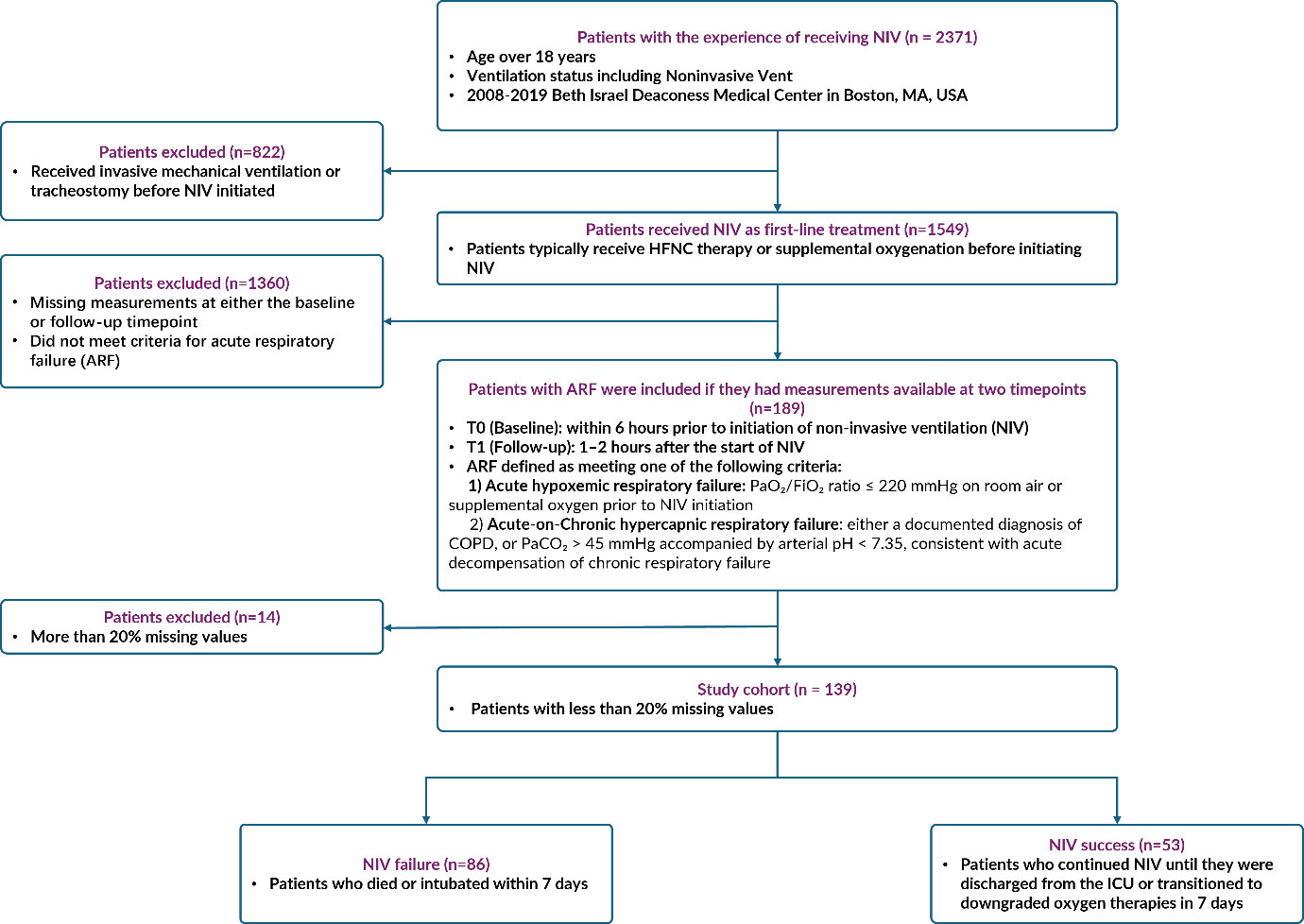


## **Fig. S2 ROC-AUC curves, Decision Curve Analysis, and calibration curves for external validation cohort**

| 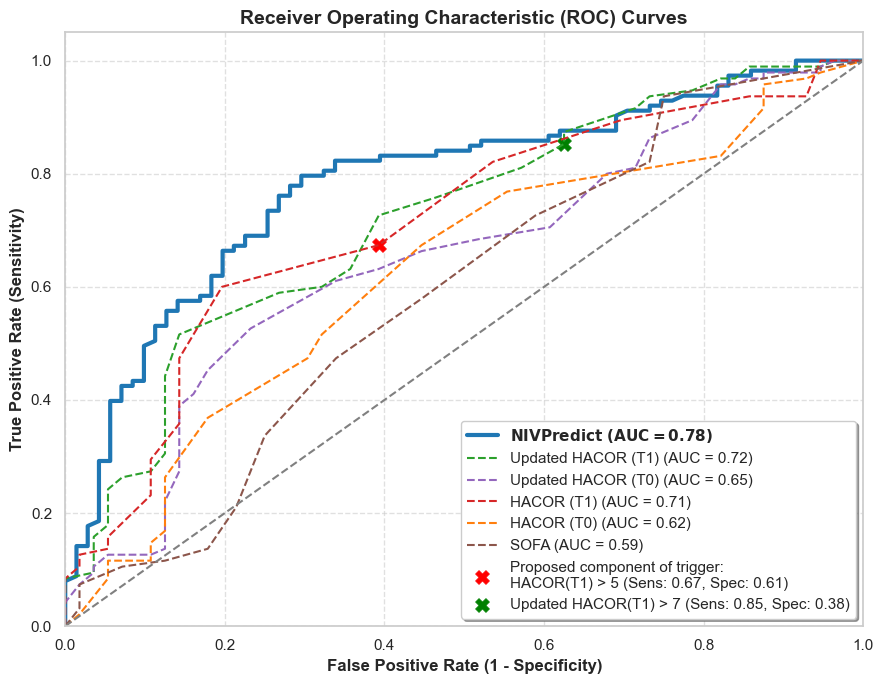 | |
| --- | --- |
| (a) | |
| 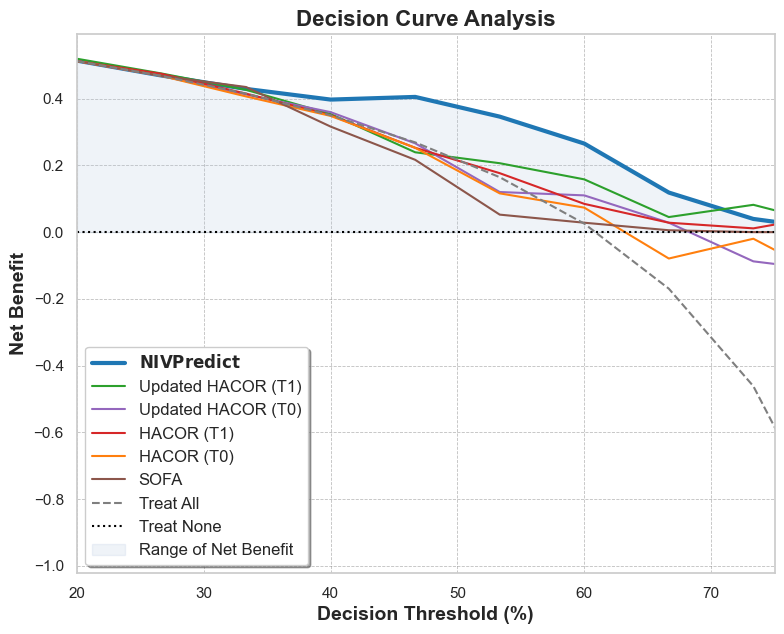 | 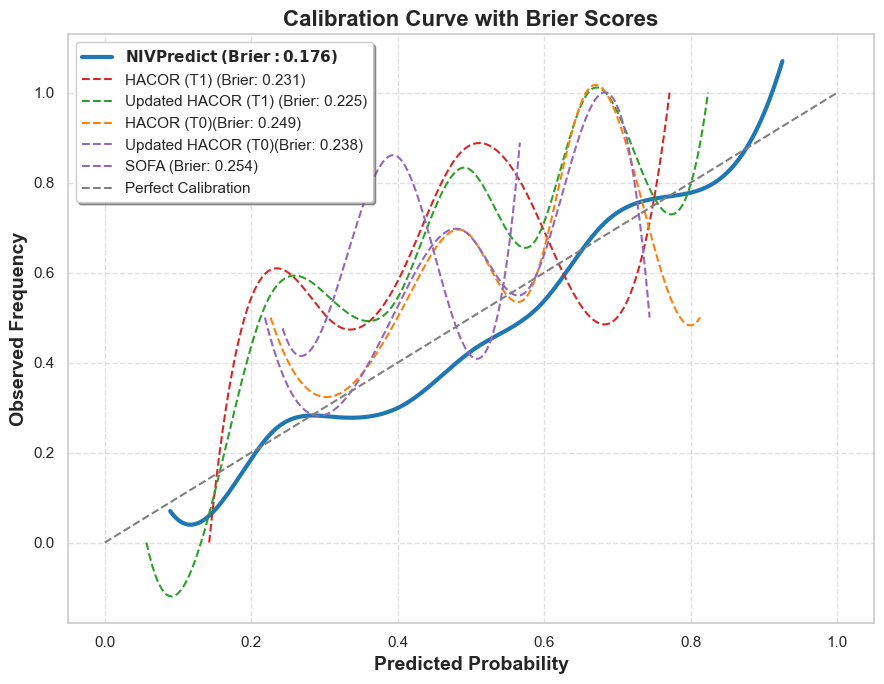 |
| (b) | (c) |

(a) The Receiver Operating Characteristic (ROC) curve comparing predictive performance in a subset of external validation where all clinical scores are available limited to 269 patients (NIV success: 137; NIV failure: 132). The HACOR/Updated HACOR threshold values proposed in original study for identifying the need for intubation after 1h of NIV initiation were applied to our external dataset for comparative analysis. (b) Decision curve analysis (DCA) in external validation.  (c) Calibration curves for the model’s predictions - a perfectly calibrated model, where the predicted probability precisely matches the observed frequencies, would follow the dashed diagonal line.

## **Fig. S3 ROC-AUC curves, Decision Curve Analysis, and calibration curves for UHNM in-hospital testing cohort**

| 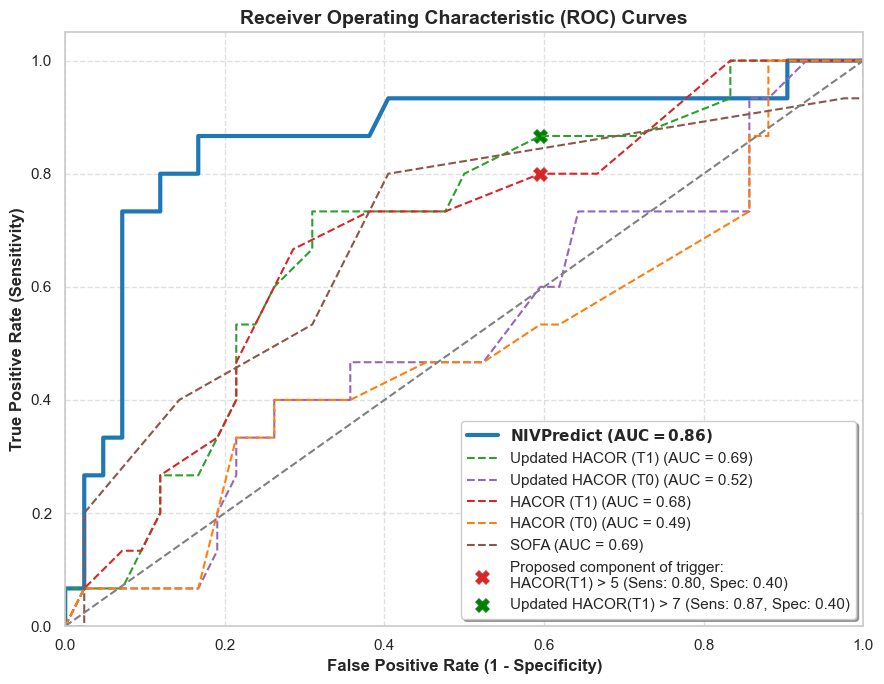 | |
| --- | --- |
| (a) | |
| 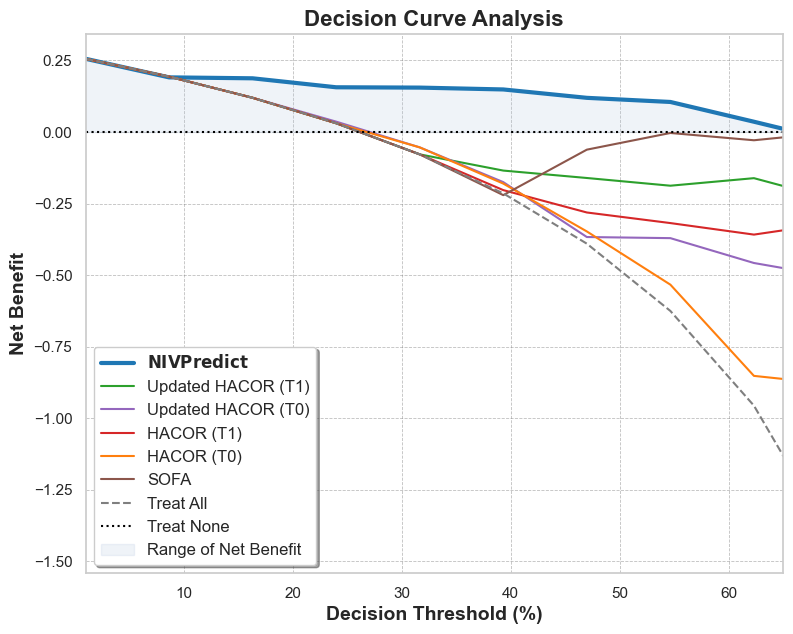 | 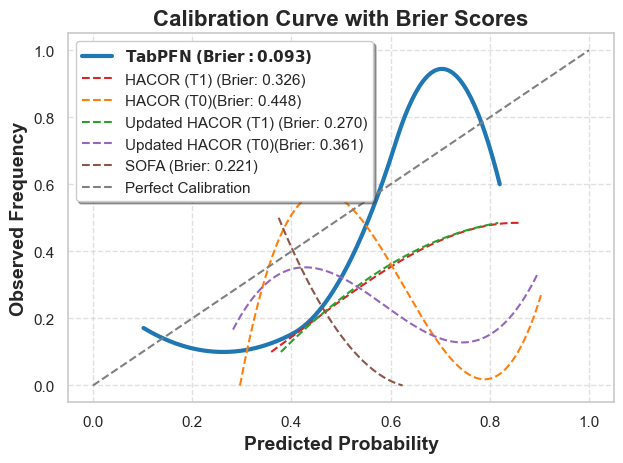 |
| (b) | (c) |

(a) The Receiver Operating Characteristic (ROC) curve comparing predictive performance in UHNM in-hospital testing cohort with 57 patients (NIV success: 42; NIV failure: 15). The HACOR/Updated HACOR threshold values proposed in original study for identifying the need for intubation after 1h of NIV initiation were applied to our external dataset for comparative analysis. (b) Decision curve analysis (DCA) in external validation.  (c) Calibration curves for the model’s predictions - a perfectly calibrated model, where the predicted probability precisely matches the observed frequencies, would follow the dashed diagonal line.

## **Fig. S4** **SHAP summary plot for the *NIVPredict* model**

| **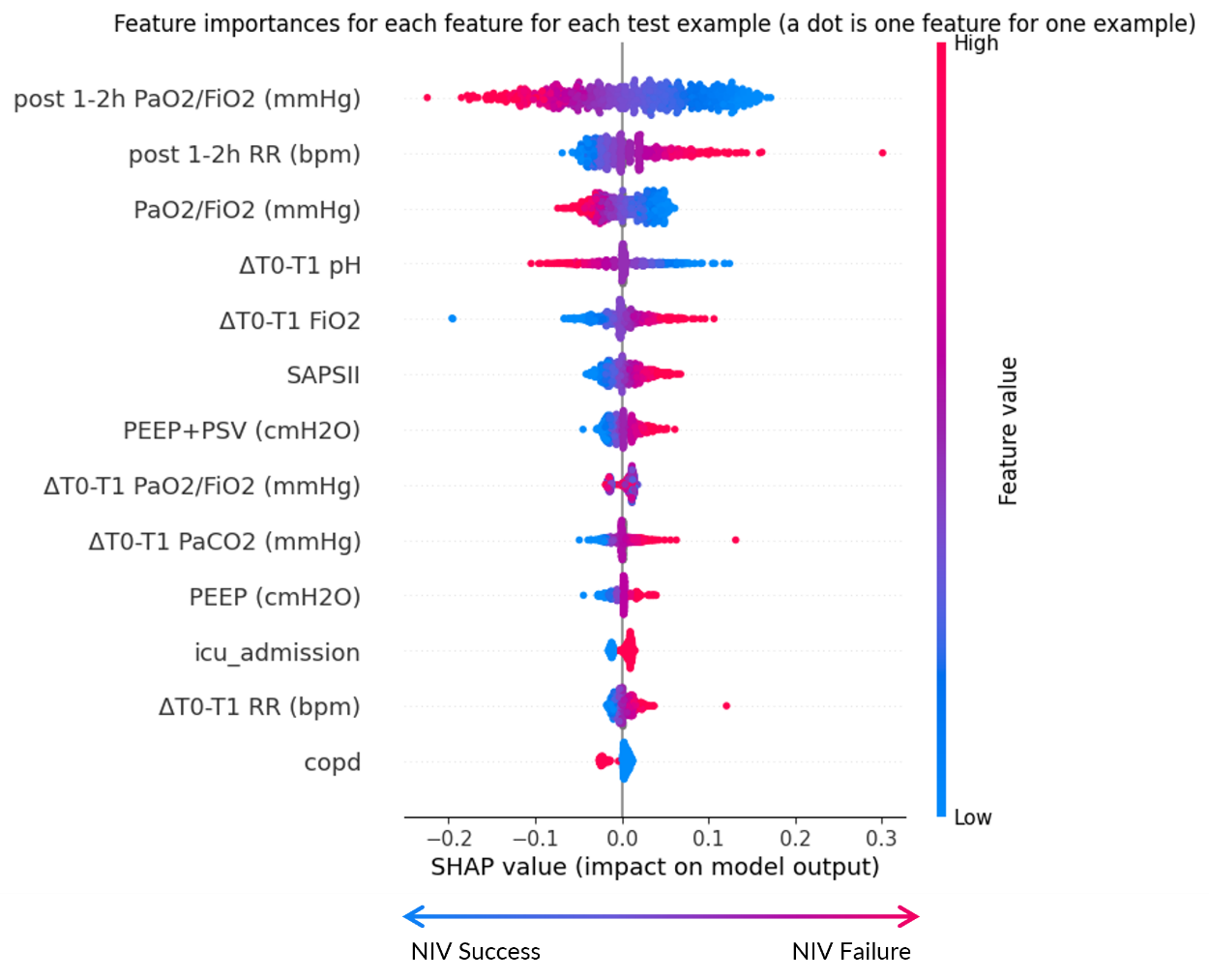** | | |
| --- | --- | --- |
| (a) | | |
| **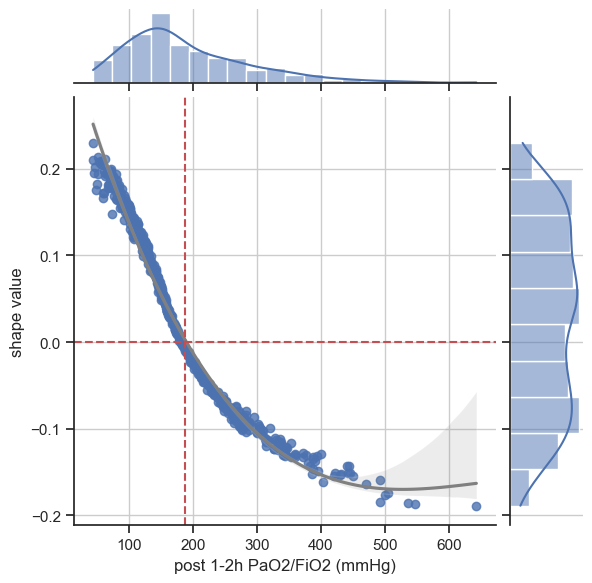** | **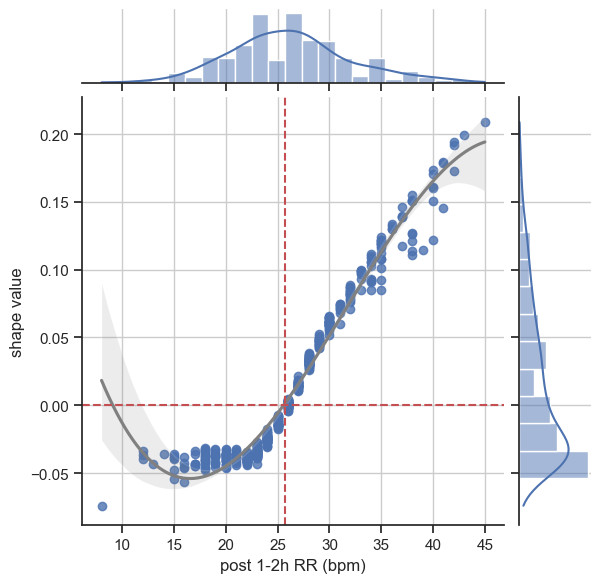** | **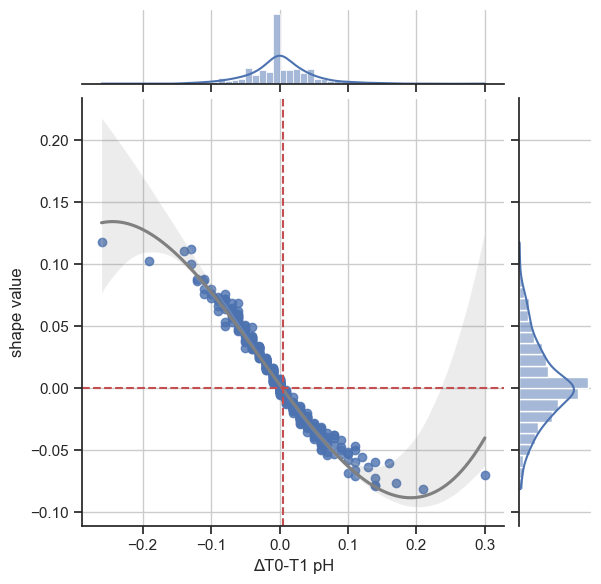** |
| (b) | | |

(a) Average SHAP values obtained from 200 iterations of repeated 5-fold cross-validation using internal training cohort. Horizontal axis: The impact of each feature on the model's prediction. Positive SHAP values indicate that the feature contributes to predicting a NIV failure, while negative SHAP values indicate a contribution to predicting NIV success. Vertical axis: The list of features used for making a prediction, ordered based on their importance, with the most important features at the top. Dots: Each dot represents a single patient in the dataset. Color gradient: Indicates the feature value for each observation, where blue represents low feature values and red represents high feature values. (b) Detailed joint distribution of selected features; the correlation between feature values and their corresponding SHAP values. Red dashed lines are drawn at the x-axis mean feature value, indicating normal level, and at the y-axis SHAP value of 0, indicating no impact on the outcome.

## **Fig. S5 SHAP feature importance plots for the NIVPredict model applied to hypoxemic and hypercapnic patient cohorts.**

| **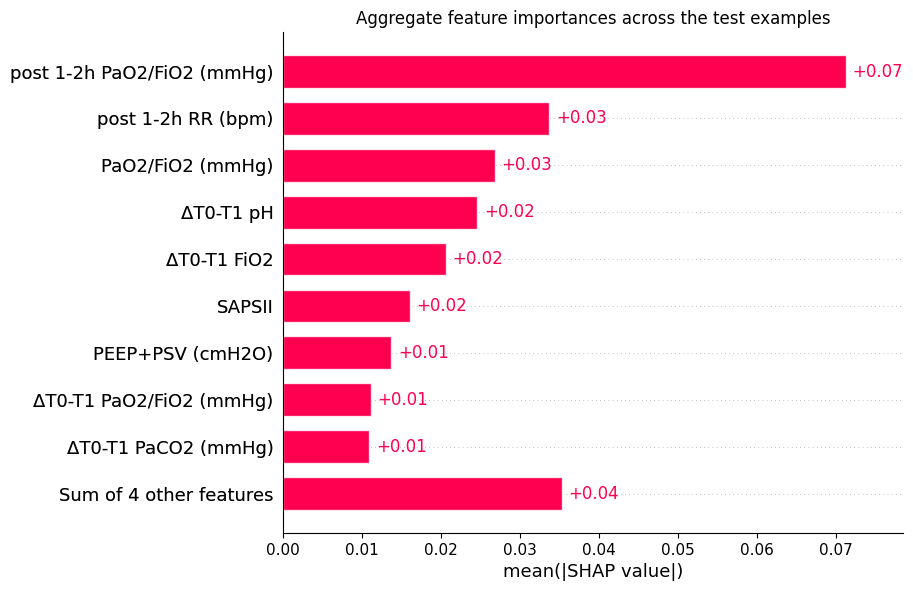** | **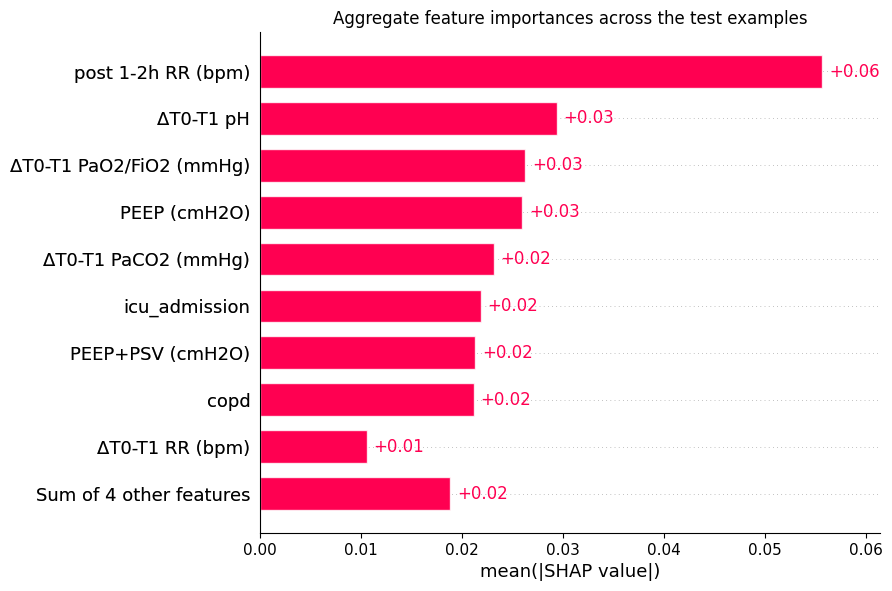** |
| --- | --- |
| Hypoxemic Cohort | Hypercapnic Cohort |
